# T lymphocyte-dependent IL-10 down-regulates a cytokine storm driven by *Toxoplasma gondii* GRA24

**DOI:** 10.1128/mbio.01455-24

**Published:** 2024-10-23

**Authors:** Claire M. Doherty, Paige R. Patterson, Julie A. Emeanuwa, Jessica Belmares Ortega, Barbara A. Fox, David J. Bzik, Eric Y. Denkers

**Affiliations:** 1 Center for Evolutionary and Theoretical Immunology and Department of Biology, University of New Mexico, Albuquerque, New Mexico, USA; 2 Department of Microbiology and Immunology, Geisel School of Medicine at Dartmouth, Lebanon, New Hampshire, USA; University of California, Irvine, Irvine, California, USA

**Keywords:** *Toxoplasma gondii*, T-cell immunity, cytokines

## Abstract

As a model organism in the study of immunity to infection, *Toxoplasma gondii* has been instrumental in establishing key principles of host anti-microbial defense and its regulation. Here, we employed an attenuated uracil auxotroph strain of Type I *Toxoplasma* designated OMP to further untangle the early immune response to this parasitic pathogen. Experiments using αβ T cell-deficient *Tcrb*^*-/-*^ mice unexpectedly revealed that an intact αβ T lymphocyte compartment was essential to survive infection with OMP. Subsequent antibody depletion and knockout mouse experiments demonstrated contributions from CD4^+^ T cells and most predominantly CD8^+^ T cells in resistance. Using transgenic knockout mice, we found only a partial requirement for IFN-γ and a lack of requirement for Toll-like receptor (TLR) adaptor MyD88 in resistance. In contrast to other studies on *Toxoplasma*, the ability to survive OMP infection did not require IL-12p40. Surprisingly, T cell-dependent IL-10 was found to be critical for survival, and deficiency of this cytokine triggered an abnormally high systemic inflammatory response. We also found that parasite molecule GRA24, a dense granule protein that triggers TLR-independent IL-12 production, acts as a virulence factor contributing to death of OMP-infected *Tcrb*^*-/-*^ and *IL-10*^*−/−*^ mice. Furthermore, resistance against OMP was restored in *Tcrb*^*-/-*^ mice via monoclonal depletion of IL-12p40, suggesting that GRA24-induced IL-12 underlies the fatal immunopathology observed. Collectively, our studies provide insight into a novel and rapidly arising T lymphocyte-dependent anti-inflammatory response to *T. gondii* which operates independently of MyD88 and IL-12 and that depends on the function of parasite-dense granule protein GRA24.

**IMPORTANCE:**

As a model infectious microbe and an important human pathogen, the apicomplexan *Toxoplasma gondii* has provided many important insights into innate and adaptive immunity to infection. We show here that a low virulence uracil auxotrophic *Toxoplasma* strain emerges as a virulent parasite in the absence of an intact T cell compartment. Both CD4^+^ and CD8^+^ T lymphocytes are required for optimal protection, in line with previous findings in other models of *Toxoplasma* infection. Nevertheless, several novel aspects of the response were identified in our study. Protection occurs independently of IL-12 and MyD88 and only partially requires IFN-γ. This is noteworthy particularly because the cytokines IL-12 and IFN-γ have previously been regarded as essential for protective immunity to *T. gondii*. Instead, we identified the anti-inflammatory effects of T cell-dependent IL-10 as the critical factor enabling host survival. The parasite dense granule protein GRA24, a host-directed mitogen-activated protein kinase activator, was identified as a major virulence factor in T cell-deficient hosts. Collectively, our results provide new and unexpected insights into host resistance to *Toxoplasma*.

## INTRODUCTION

*Toxoplasma gondii* is a protozoan parasite belonging to the apicomplexan phylum that includes other human pathogens such as *Plasmodium* and *Cryptosporidium*. Together this family of infectious agents is responsible for the illness and death of millions each year. *Toxoplasma* itself is estimated to be present in over a third of the global population and is the second leading cause of foodborne death in the United States (1, 2). In immunocompetent individuals, *T. gondii* establishes a latent infection within the central nervous system, where it remains for the lifespan of the host (3). However, in certain immunocompromised populations, the most common being HIV-AIDS patients, *T. gondii* may become a life-threatening infection (4–6). Congenital infection with *Toxoplasma* is also a serious concern. In these cases, there are a range of possible pre- and post-natal outcomes, including hydrocephaly, blindness, mental incapacitation, and death (7, 8).

Over the last two or more decades, mouse models of immunity have revealed that initiation of host defense against *T. gondii* depends to a great extent upon TLR11/12-mediated recognition of parasite profilin, which signals through MyD88 to initiate IL-12 production in dendritic cells (DCs) (9–16). Studies using C57BL/6 background *batf3^−/−^* animals indicate that CD8α^+^ and CD103^+^ conventional DC (cDC) are a key source of IL-12 that fuels the emergence of protective IFN-γ-producing CD8^+^ T cells (12). Nevertheless, recent work suggests that infection with the *Toxoplasma* uracil auxotroph cps1-1 induces CD8^+^ T cell immunity driven by cDC that operates independently of IL-12 (17). It has also been found that CD4^+^ Th1 cells emerge as the dominant protective T cell subset in the absence of BATF3 during infection with an engineered *Toxoplasma* strain lacking the virulence factor ROP5 (18). IL-12-dependent NK cell activation provides an important innate source of IFN-γ that is particularly important during the early stages of infection prior to the appearance of activated effector T cells (19, 20). More recently, innate lymphoid cells have been discovered as an important component of the early protective response to *Toxoplasma* (21–27).

IFN-γ itself confers protection through the induction of immunity-related GTPase (IRG) proteins that mediate the destruction of the parasitophorous vacuole membrane in infected cells (28–33), as well as through other mechanisms including tryptophan starvation, production of nitric oxide, and induction of the respiratory burst (34–39). Inflammatory Gr-1^+^ monocytes are regarded as major effectors of IFN-γ-dependent killing of *Toxoplasma* in both the peritoneal cavity and intestinal mucosa following infection (40–42). Other effector cells such as neutrophils may also contribute to protection through mechanisms such as release of neutrophil extracellular traps (43, 44). The inflammatory Th1 program is curbed by the regulatory effects of IL-10, a cytokine previously shown to be produced by Th1 cells themselves during *T. gondii* infection (45–47).

Engineered uracil auxotrophic strains of *Toxoplasma* have provided important insights into the induction of mouse adaptive immunity to the parasite. These mutant strains are derived from Type I strain RH tachyzoites, virulent parasites that widely disseminate following infection, inducing overproduction of inflammatory cytokines and culminating in host death within 10–14 days (48, 49). The prototypic cps1-1 uracil auxotroph invades cells normally but fails to replicate and/or persist without an exogenous source of uracil *in vitro* and does not persist *in vivo* (50). Intraperitoneal inoculation of cps1-1 triggers robust Th1-based protective immunity to challenge with RH strain tachyzoites and vaccination with this uracil auxotroph also protects mice against infection with lower virulence cyst-forming *Toxoplasma* strains (51, 52). The response induced by cps1-1 is marked by local production of IL-12 and IFN-γ, along with the occurrence of antigen-specific CD8^+^ T cells that appear rapidly following infection in partial dependence upon CD4^+^ T lymphocytes (51, 53). The CD8^+^ T cells elicited by cps1-1 express an effector (CD62L^low^KLRG1^+^ CD127^low^) phenotype, and they persist for up to 70 days post-inoculation (54). The use of uracil auxotrophic *T. gondii* has revealed that immunity can be generated independently of MyD88 in a mechanism involving IL-12 induction by parasite-dense granule protein GRA24 (55). Furthermore, uracil auxotrophic *Toxoplasma* strains have been shown to elicit highly effective anti-tumor responses in mouse models of melanoma, pancreatic, and ovarian cancer (56–60). At least part of this activity is attributable to the targeted invasion of macrophages and dendritic cells stimulating upregulation of costimulatory molecules CD80/CD86 and increased production of IL-12 (60).

The parasite strain OMP is a more recently developed uracil auxotroph that also elicits a protective immune response to *Toxoplasma* (61). Deletion of the gene orotidine-5’-monophosphate decarboxylase combined with the deletion of the critical pyrimidine salvage enzyme uridine phosphorylase renders OMP into a nonreverting pyrimidine auxotroph of *Toxoplasma*. The OMP parasite has been employed to investigate adaptive immune responses to *T. gondii* (55, 61). Here, we report the unexpected discovery that mouse resistance to OMP requires an intact T cell compartment. Protection in this model occurs independently of IL-12, MyD88, and Gr-1^+^ effector monocytes and only partially depends upon IFN-γ. In marked contrast, T cell-dependent IL-10 was an essential factor in surviving OMP infection. In T cell-deficient hosts, the parasite-dense granule protein GRA24 emerges as a virulence factor driving proinflammatory cytokine overproduction and early death. Collectively, these data reveal unexpected and previously unrecognized facets of the immune response to *Toxoplasma*.

## RESULTS

### Protective immunity to the attenuated *T. gondii* uracil auxotroph OMP requires Rag1-dependent cells

**Fig 1:**
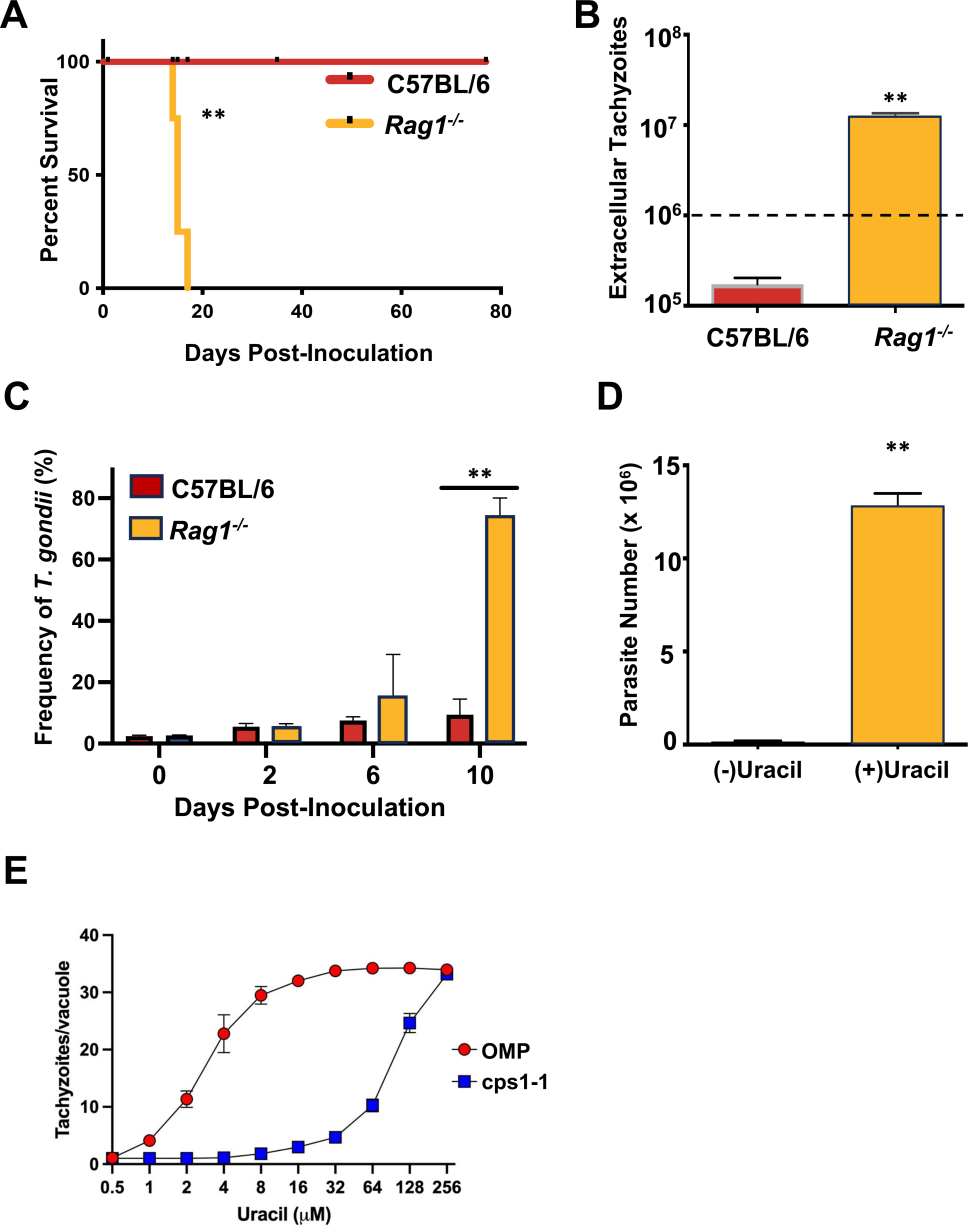
Control of OMP requires an intact lymphocyte compartment. (**A**) Susceptibility of *Rag1^−/−^* relative to C57BL/6 mice following i. p. inoculation of 10^6^ OMP tachyzoites. (**B**) Numbers of OMP tachyzoites in the peritoneal cavities of WT and KO mice 12 days post-infection. Dotted line indicates infecting dose. (**C**) Flow cytometric analysis of OMP tachyzoite frequency in the peritoneal cavity following infection in *Rag1^−/−^* and C57BL/6 mice. (**D**) Requirement for exogenous uracil during *in vitro* culture of OMP tachyzoites isolated from *Rag1^−/−^* mice. Peritoneal tachyzoites from 12-day infected mice were inoculated onto human fibroblast monolayers with and without exogenous uracil (300 µM), and parasites were enumerated 3 days later. In these collective experiments, mice (*n* = 4 per group) were infected by i. p. inoculation with 10^6^ OMP tachyzoites. (**E**) OMP and cps1-1 uracil growth rescue profiles. Intracellular replication rate was measured using duplicate cultures in three independent experiments. Data points show the average number of tachyzoites/vacuole (±SEM). Susceptibility studies were repeated in three independent experiments. Ex vivo studies were repeated in two independent experiments. Susceptibility study statistics were acquired using Kaplan Meier log-rank tests to analyze the data where **P < 0.01. Un-paired student’s *t*-test with Welch’s correction and multiple unpaired *t*-tests were used to analyze the data where **P < 0.01.

The uracil auxotrophic strain OMP elicits robust protective T cell immunity in C57BL/6 wild-type (WT) mice. Surprisingly, we found that lymphocyte cell-deficient *Rag1^−/−^* knockout (KO) mice uniformly succumbed to OMP infection 14–21 days post-intraperitoneal (i. p.) inoculation relative to *Rag1^+/+^* animals (Fig. 1A). In parallel experiments, collection of peritoneal exudate cells (PECs) at day 12 post-infection revealed expansion of extracellular tachyzoites in KO mice and contraction of parasite number in WT mice (Fig. 1B). Using flow cytometry to measure extracellular tachyzoite frequency over time revealed expansion of the OMP tachyzoite population occurring between 6 and 10 days post-infection in the *Rag1^−/−^* mice (Fig. 1C). We considered the possibility that we had somehow selected for revertant parasites in the Rag1 KO mice, or our original inoculum was contaminated with WT parasites. However, neither proved to be the case as tachyzoites isolated from the peritoneal cavity of day 12 infected *Rag1^−/−^* mice retained a strict requirement for exogenous uracil when placed into culture on fibroblast monolayers (Fig. 1D). While the ~10-fold expansion of OMP in the peritoneal cavity of *Rag1^−/−^* mice compared with the fivefold contraction in C57BL/6 mice was surprising (Fig. 1B), these results correlated with survival data showing that *Rag1^−/−^* mice succumbed to OMP, whereas C57BL/6 mice did not (Fig. 1A). Seemingly paradoxically, previous results have shown that *Rag1^−/−^* mice survive high-dose infection with the uracil auxotroph cps1-1 (62). Consequently, we measured the uracil auxotroph replication rescue profiles of cps1-1 compared to OMP. Detectable replication of OMP was rescued by relatively low doses of uracil (~1 µM), whereas similar detectable replication rescue of cps1-1 required ~32 µM uracil (Fig. 1E). Similarly, full rescue of OMP replication required ~16 µM uracil, whereas full rescue of cps1-1 replication required ~256 µM uracil. Together, these results suggest that a lack of clearance of OMP parasites in *Rag1^−/−^* mice ultimately promoted a ~10-fold expansion of OMP parasites in the peritoneal cavity after 12 days of infection due to the ability of OMP to replicate at a very slow rate in low uracil concentration environments. We note that the net 10-fold expansion we observed in the peritoneal cavity of *Rag1^−/−^* mice (Fig. 1B) was a relatively modest increase, given that this correlates with only 3–4 parasite divisions over the 12-day course of the experiment.

### Inoculation with OMP induces an influx of CD4^+^ and CD8^+^ T lymphocytes in the peritoneal cavity which are required for protection

**Fig 2:**
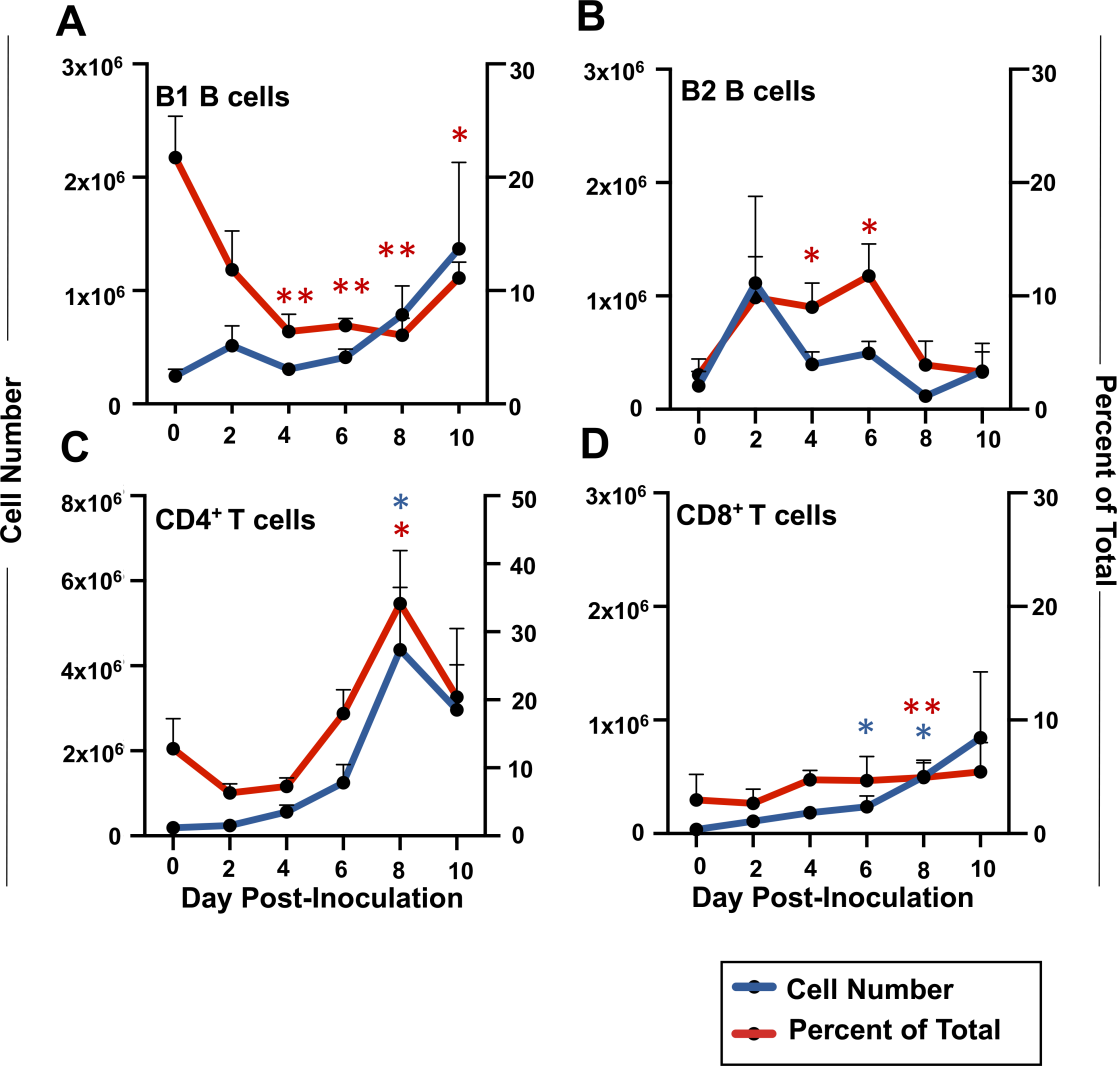
Flow cytometric profiling of cells in the peritoneal cavity reveals an influx of T lymphocytes 6–8 days post-infection with OMP in C57BL/6 animals. Cell number (blue line; left y-axis) and percentage (red line; right y-axis) of (**A**) B1 B cells (B220^lo^ CD11b^+^ CD5^−^), (**B**) B2 B cells (B220^hi^ CD11b^−^ CD5^−^), (**C**) CD4^+^ T lymphocytes (TCRβ^+^CD4^+^), and (**D**) CD8^+^ T lymphocytes (TCRβ^+^CD8^+^) following inoculation with 10^6^ OMP. Data from three independent infections (*n* = 2) with average and SEM displayed. Statistics were acquired through unpaired Student *t*-tests relative to day 0 samples with *P < 0.05 and **P < 0.01. Blue and red asterisks, significance of cell number and percentage data, respectively.

We next assessed the status of Rag1-dependent cells over the course of 10 days following OMP inoculation in WT mice. As expected for the peritoneal cavity (63), there was a notable population of B220^lo^CD11b^+^CD5^+^ B1 B cells. The percentage of these cells dropped over the course of infection, but absolute numbers stayed relatively constant (Fig. 2A, blue and red lines, respectively). The percentage of conventional B2 cells (B220^hi^CD11b^−^CD5^−^) was significantly increased at days 4 and 6, although total numbers remained relatively stable over the course of infection (Fig. 2B). In contrast to these small effects, we observed a striking increase in CD4^+^ T cells occurring between days 4 and 8 after OMP inoculation (Fig. 2C). We found a small increase in CD8^+^ T lymphocytes over the time course of infection (Fig. 2D).

**Fig 3:**
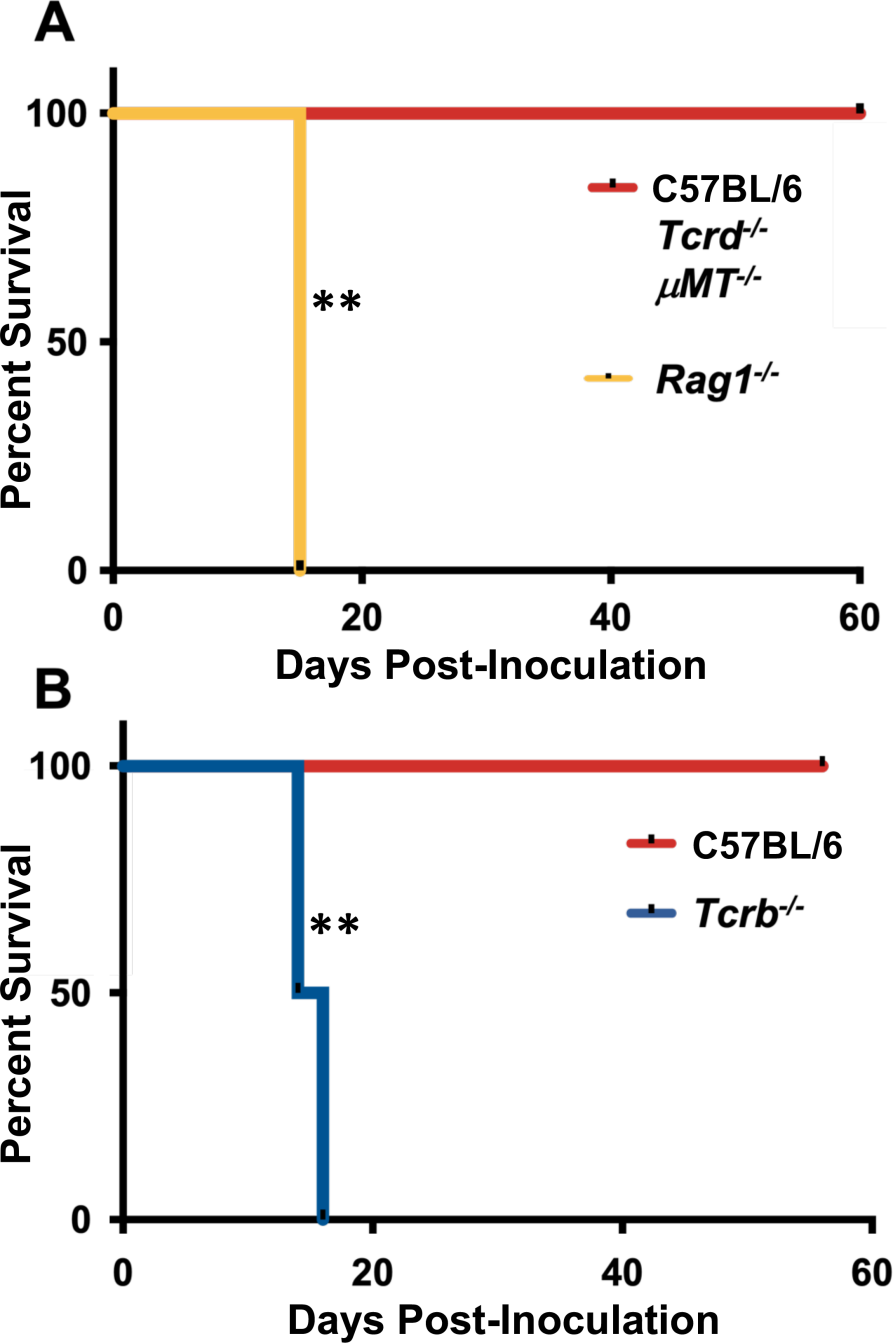
TCRβ-expressing T cells are required to resist OMP infection. Mice were infected by i. p. injection of 10^6^ OMP tachyzoites. (**A**) γδT cell-deficient (*Tcrd^−/−^*) deficient mice as well as B cell-deficient (*μMT*) mice were infected and survival compared to *Rag1^−/−^* animals (*n* = 4 per strain). (**B**) Survival of mice deficient in αβ T lymphocytes (*Tcrb^−/−^*) relative to C57BL/6 controls (one representative experiment of four independent experiments; *n* = 4 per strain). Susceptibility study statistics were acquired using Kaplan Meier log-rank tests to analyze the data where **P < 0.01.

We employed a transgenic KO approach to determine the importance of each Rag-dependent cell population in protection. *Tcrd^−/−^* and *μMT^−/−^* mice displayed an identical resistance phenotype to WT mice, ruling out γδ T lymphocytes and B lineage cells, respectively, in protection against OMP parasites (Fig. 3A). In contrast, infection of αβ T cell KO mice (*Tcrb^−/−^*) resulted in a susceptibility phenotype virtually identical to that of the Rag1 KO animals (Fig. 3B).

**Fig 4:**
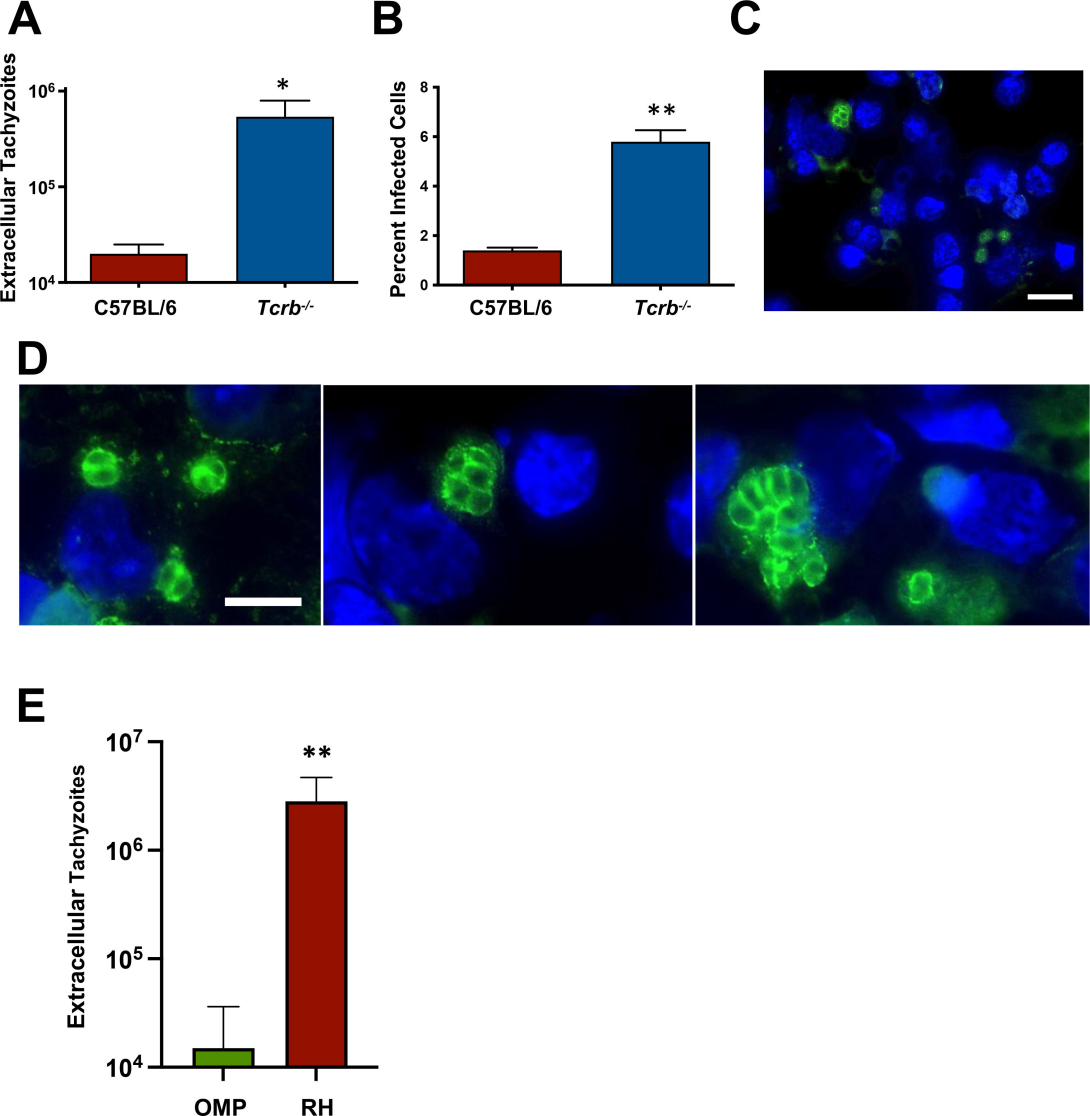
OMP persistence and survival in *Tcrb^−/−^* mice. (**A–D**) C57BL/6 and *Tcrb^−/−^* mice (*n* = 4 per strain) were infected with 10^6^ OMP tachyzoites, then peritoneal cavity washout cells were collected at day 12 post-infection for evaluation. (**A**) Extracellular tachyzoite number in the peritoneal cavity. (**B**) Percent infected cells in the peritoneal washout preparations, calculated from three fields of view containing approximately 100 cells each. (**C**) Representative immunofluorescence microscopy image showing approximately 5% infection rate in *Tcrb^−/−^* mice. Scale bar, 20 µm. (**D**) Three close-up images of OMP-infected cells in *Tcrb^−/−^* mice. Scale bar in left image, 10 µm. (**E**) Extracellular tachyzoite numbers in the peritoneal cavity of *Tcrb^−/−^* mice 6 days after infection with 10^4^ OMP and RH parasites (*n* = 4 mice per parasite strain). Statistical significance was evaluated using an unpaired two-tailed Mann-Whitney test (**A**), an unpaired two-tailed Student *t*-test (**B**), and an unpaired two-tailed Student’s *t*-test (**D**) where *P < 0.05 and **P < 0.001. Data are representative of two independent experiments.

To determine the status of OMP parasites in *Tcrb^−/−^* mice, we collected peritoneal washouts from WT and Tcrb KO mice 12 days after inoculation of 10^6^ tachyzoites. OMP extracellular tachyzoites (Fig. 4A) and percent infected cells (Fig. 4B) were low in C57BL/6 mice. In *Tcrb^−/−^* animals, levels of extracellular parasites were significantly increased compared to WT mice. Nevertheless, they remained at or slightly below the inoculating dose of 10^6^ (Fig. 4A). Along similar lines, the percent infection, while significantly increased in the *Tcrb^−/−^* compared to WT peritoneal cells, was relatively low at approximately 5% (Fig. 4B and C). Of the cells that were infected in the KO mice, we found clear evidence that the parasites were able to replicate, at least to a limited extent (Fig. 4D). To compare this to infection with RH, which is the virulent parental parasite parental strain of OMP, we infected *Tcrb^−/−^* mice with a low number (10^4^) of OMP and RH, then enumerated parasites 6 days later. As shown in Fig. 4E, extracellular RH tachyzoites expanded in number by approximately 500-fold, whereas OMP parasites remained at close to the inoculating dose. Overall, we conclude that while OMP parasites can persist and replicate to a limited extent in *Tcrb^−/−^* mice, they remain attenuated in this T cell-deficient setting. This suggested that uncontrolled parasite replication may not be the sole cause of death in mice lacking T cells.

**Fig 5:**
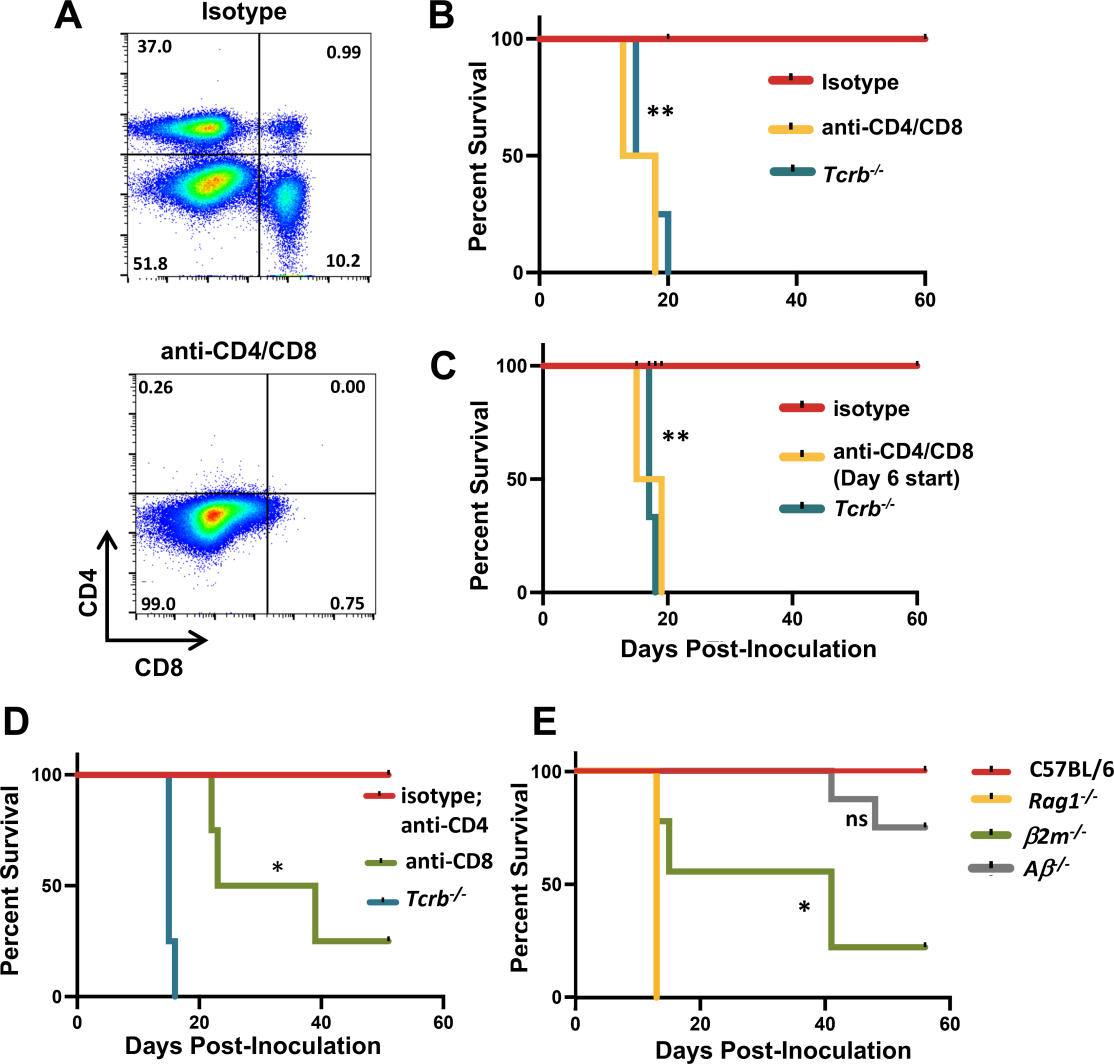
CD4^+^ and CD8^+^ T lymphocytes act together to provide optimal resistance to OMP infection. (**A**) Presence of peritoneal cavity T cells following administration of anti-CD4 and anti-CD8 mAb. Flow cytometric analysis was conducted 2 days following OMP inoculation. Susceptibility of OMP-infected mice following CD4^+^ and CD8^+^ T cell depletion starting prior to OMP infection (**B**) as well as 6-days post inoculation (**C**). In panel (**D**), mice were treated with anti-CD4 and anti-CD8 mAb separately, and survival was assessed following OMP infection. (**E**) Susceptibility studies on mice deficient in MHC class I (*β2m^−/−^*) and MHC class II molecules (*Aβ−/−*). Experiments were repeated three times (*n* = 4). Susceptibility study statistics were acquired using Kaplan Meier log-rank tests to analyze the data where *P < 0.05 and **P < 0.01.

To determine the importance of CD4^+^ and CD8^+^ T cells in resistance, we used a mAb depletion approach to eliminate each respective T cell subset (Fig. 5A). As predicted, co-administration of anti-CD4 and anti-CD8 mAb resulted in susceptibility to OMP (Fig. 5B). Depletion of T cells initiated at day 6 post-OMP infection also resulted in extreme susceptibility, a result that implicates the day 6 T cell increase in protection against OMP (Fig. 5C).

The relative contributions of CD4^+^ and CD8^+^ T cells to protection were next assessed by depleting cells separately with mAb. Depletion of CD8^+^ T cells alone resulted in partial susceptibility, whereas depletion of CD4^+^ T cells had no discernable effect on ability to resist OMP (Fig. 5D). We also used MHC class I KO (*β2m^−/−^*, CD8^+^ T cell deficient) and MHC class II KO (*Aβ^−/−^*, CD4^+^ T cell deficient) mice to assess the contribution of CD4^+^ and CD8^+^ T cells to protection. The results were overall similar to the mAb depletion experiments, insofar as class I-deficient mice displayed a partial susceptibility phenotype closely resembling mice given anti-CD8 mAb (Fig. 5E). For the case of MHC class II KO mice, we detected a small increase in susceptibility to OMP. Failure to detect this subtle increase in susceptibility in anti-CD4 mAb-treated mice could be due to incomplete depletion of CD4^+^ T lymphocytes. Regardless, the collective results in Fig. 5 argue that CD8^+^ T cells are the major mediators of protection against OMP. The failure of CD8^+^ depletion (either through mAb depletion or β2-microglobulin gene deletion) to recapitulate the extreme susceptibility of the *Tcrb^−/−^* mice argues for a secondary contribution of CD4^+^ T cells in resistance to this uracil auxotroph.

**Fig 6:**
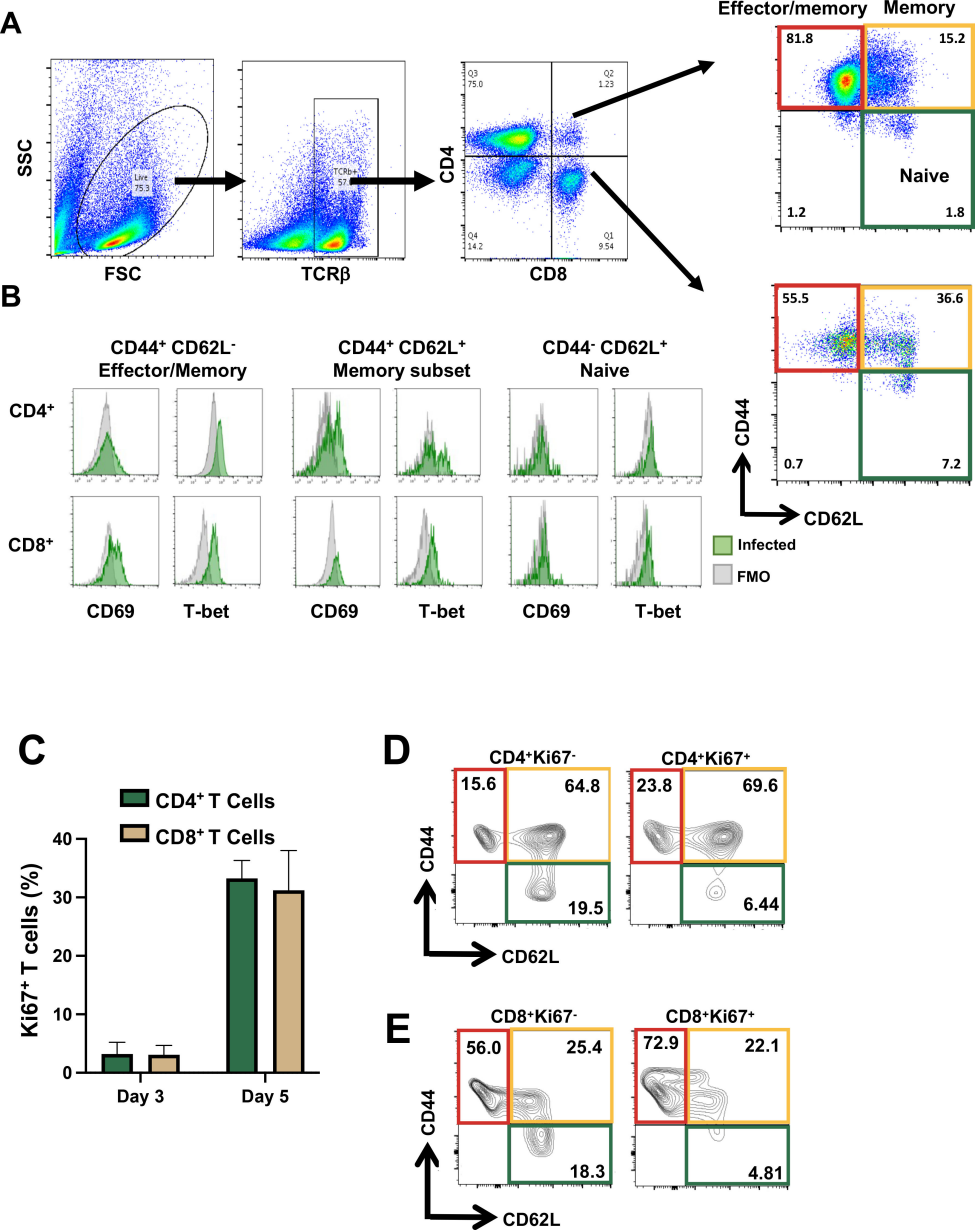
Phenotypic profile of T lymphocytes elicited by OMP indicates a predominant-activated effector/memory phenotype. C57BL/6 mice were inoculated with 10^6^ OMP tachyzoites, then 6 days later PEC were collected for flow cytometric analysis. (**A**) Flow gating scheme to identify effector/memory (CD44^+^CD62L^−^), memory (CD44^+^CD62L^+^), and naïve subsets (CD44^−^CD62L^+^) of CD4^+^ T cells and CD8^+^ T cells. (**B**) Expression of CD69 and T-bet on subsets of CD4^+^ and CD8^+^ T cells. (**C**) Expression of Ki67 on peritoneal T cells assessed 3 and 5 days post-OMP infection. (**D**) CD4^+^ T cell expression of CD44 and CD62L amongst Ki67-negative (left) and Ki67-positive (right) populations assessed 5 days post-OMP infection. (**E**) As in D, gated on CD8^+^ T lymphocytes. FMO, fluorescence-minus-one control. One representative experiment of three independent studies (*n* = 3 mice per infection) displayed.

Given the requirement for T cells in protection, we used multi-color flow cytometry to examine their activation status in the peritoneal cavity. Six days post OMP inoculation of WT mice, PEC were isolated, T cells were identified based upon expression of the T cell receptor β chain, and CD4 vs CD8 cells were gated for determination of CD44 and CD62L expression (Fig. 6A). Within both CD4 and CD8 subsets, we found that effector/memory (CD44^hi^CD62L^lo^) cells predominated, with intermediate levels of memory (CD44^hi^CD62L^hi^) and very low levels of naive (CD44^lo^CD62L^hi^) cells present. Within these populations, we assessed the expression of the activation marker CD69 as well as the Th1 and CD8 effector transcription factor T-bet (Fig. 6B). Both CD69 and T-bet were upregulated in effector/memory and memory subsets of CD4^+^ and CD8^+^ T cells. As expected, neither of these molecules were expressed to high degree in naive phenotype cells. We also examined naive/effector/memory of splenic T cells isolated from OMP-infected mice. In this case (Fig. S1), both CD4^+^ and CD8^+^ T lymphocytes displayed a predominant naive (CD44^lo^CD62L^hi^) phenotype in contrast to peritoneal cavity T cells.

Kinetic studies of the immune response to OMP revealed an increase in T cell number at 6 days following infection (Fig. 2C and D). To examine whether cell proliferation contributed to this increase, we examined the nuclear expression of Ki67, a marker of active cell cycling. While approximately 5% of CD4^+^ and CD8^+^ T cells expressed Ki67 at 3 days post-infection, the percent expression increased markedly to 30% at 5 days post-OMP inoculation (Fig. 6C).

Of the proliferating (Ki67^+^) CD4^+^ T cells, the memory subset (CD44^hi^CD62L^hi^) was the most predominant at day 5 post inoculation followed by effector/memory cells (CD44^hi^CD62L^lo^; Fig. 5D, upper right). Approximately 20% of Ki67^−^ CD4^+^ T cells expressed a naive (CD44^lo^CD62L^hi^) phenotype (Fig. 6D, upper left). For CD8^+^ T lymphocytes, over 70% of proliferating cells expressed an effector/memory phenotype (CD44^hi^CD62L^lo^), and most of the remaining cells fell into the memory subset (CD44^hi^CD62L^hi^; Fig. 6E, lower right). Similar to the CD4^+^ T cells, approximately 20% of non-proliferating Ki67^−^ CD8^+^ cells displayed a naive phenotype (Fig. 6E, lower left). Together, the results show that T cells induced by OMP are actively cycling and overall express and effector or effector/memory phenotype, consistent with their protective activity.

### T cell-mediated immunity to OMP operates independently of IL-12, only partially depends upon IFN-γ, and does not require Ly6C/G^+^ effector cells

**Fig 7:**
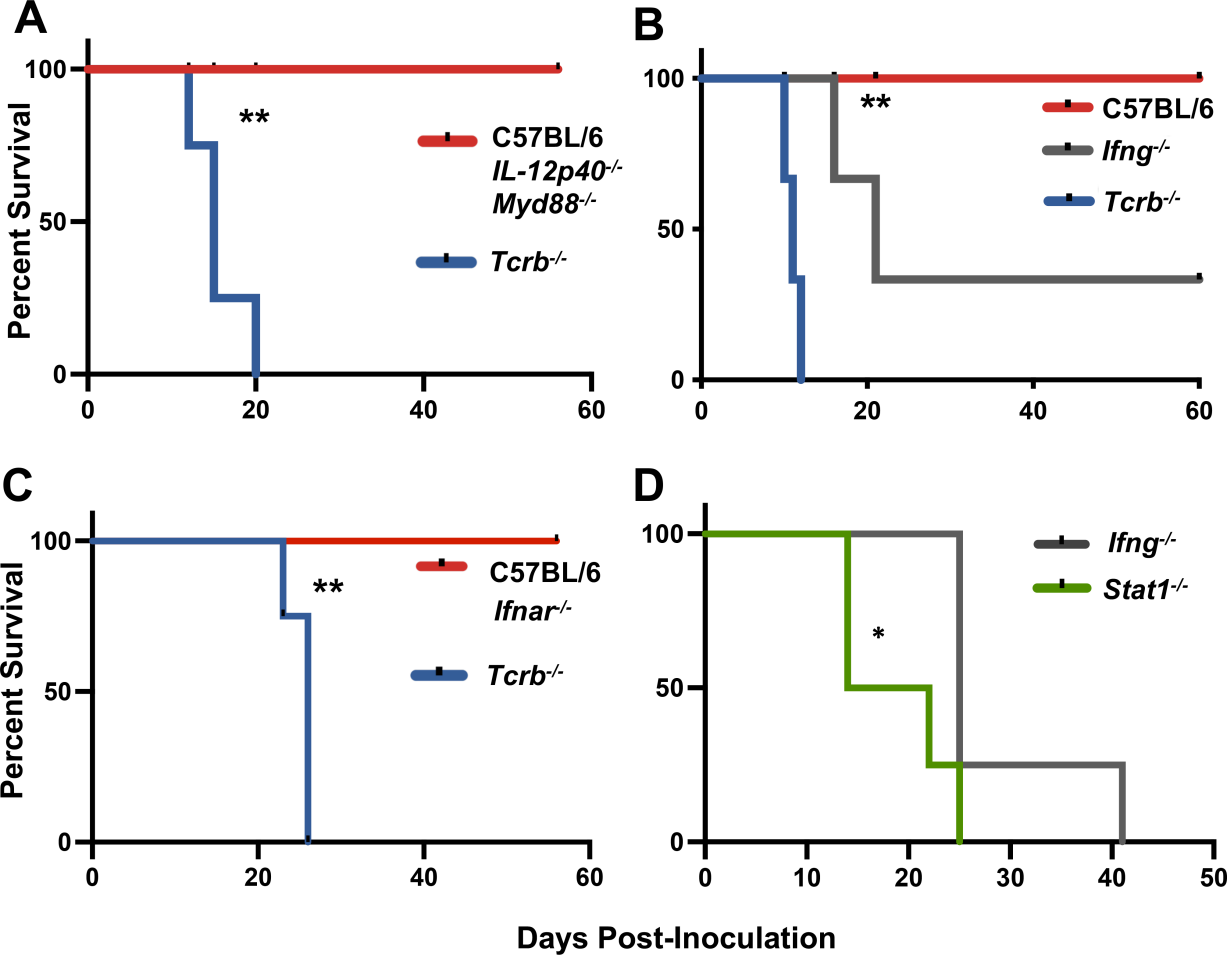
Control of OMP requires neither MyD88 nor IL-12p40 and only partially involves IFN-γ. OMP parasites were inoculated into (**A**) C57BL/6, *IL-12p40^−/−^*, *Myd88^−/−^*, and *Tcrb^−/−^* mice via i. p. injection and survival was monitored. (**B**) OMP tachyzoites were inoculated into C57BL/6, *IFN-γ^−/−^*, and *Tcrb^−/−^* and survival monitored. (**C**) Survival of *Ifnar^−/−^ and Tcrb^−/−^* after inoculation with OMP. (**D**) Comparison of susceptibility in *Stat1*^−/−^ and *Ifng^−/−^* mice. Susceptibility studies were independently repeated three times (**A and B**), one time (**C**), and two times (**D**) with *n* = 4 mice per strain. Susceptibility study statistics were acquired using Kaplan Meier log-rank tests to analyze the data where *P < 0.05 and **P < 0.01.

Next, we wanted to gain insight into the requirements for the rapid generation and function of T cells triggered by OMP. Numerous studies extending back two decades or more have argued for IL-12 as an essential mediator in generation of protective T cell resistance to *Toxoplasma* (35, 64–67). Therefore, we were greatly surprised to find that *IL12p40^−/−^* mice maintained full resistance to OMP, unlike *Tcrb^−/−^* animals (Fig. 7A). It is also noteworthy that *Myd88^−/−^* mice survived infection, a result that is in line with the known existence of MyD88-independent immunity during *T. gondii* infection (Fig. 7A) (55, 68, 69). Thus, the protective T cell response to OMP is elicited independently of MyD88 and, even more significantly, does not rely on IL-12.

The cytokine IFN-γ is known as the central mediator of resistance to *T. gondii*, acting through diverse mechanisms including induction of immunity-related GTPases, inducible nitric oxide synthase, and indolamine dioxygenase (35, 70–72). Known sources of IFN-γ during *Toxoplasma* infection include both CD4^+^ and CD8^+^ effector cells (73, 74). We, therefore, assessed the role of IFN-γ in resistance to OMP. Although *Ifng^−/−^* mice did indeed display increased susceptibility to OMP, the animals were nevertheless significantly more resistant relative to the T cell-deficient animals (Fig. 7B). Thus, while IFN-γ plays a role, it is not a major mediator of resistance during OMP infection.

Despite the dominant role of IFN-γ in resistance to wild-type *T. gondii* infection, Type I IFN production has also been reported during infection (75). We considered the possibility that Type I IFN played a role in resistance to OMP, but we found that IFNAR KO mice that lack the Type I IFN receptor were resistant to OMP (Fig. 7C). Given that mice lacking the Type I IFN receptor retain IFN-γ responsiveness, we used *Stat1^−/−^* animals to interrogate the role of STAT1-mediated type I and type II IFN signaling in protection against OMP. Interestingly, we found that STAT1 deficiency resulted in increased susceptibility to infection compared to deficiency in IFNγ alone (Fig. 7D). This result suggests that both Type I and Type II IFN may contribute to resistance to OMP. Nevertheless, this conclusion must be interpreted with caution since a recent study provided evidence that STAT1 signaling is required in T cells to instruct the production of IL-10 (47).

**Fig 8:**
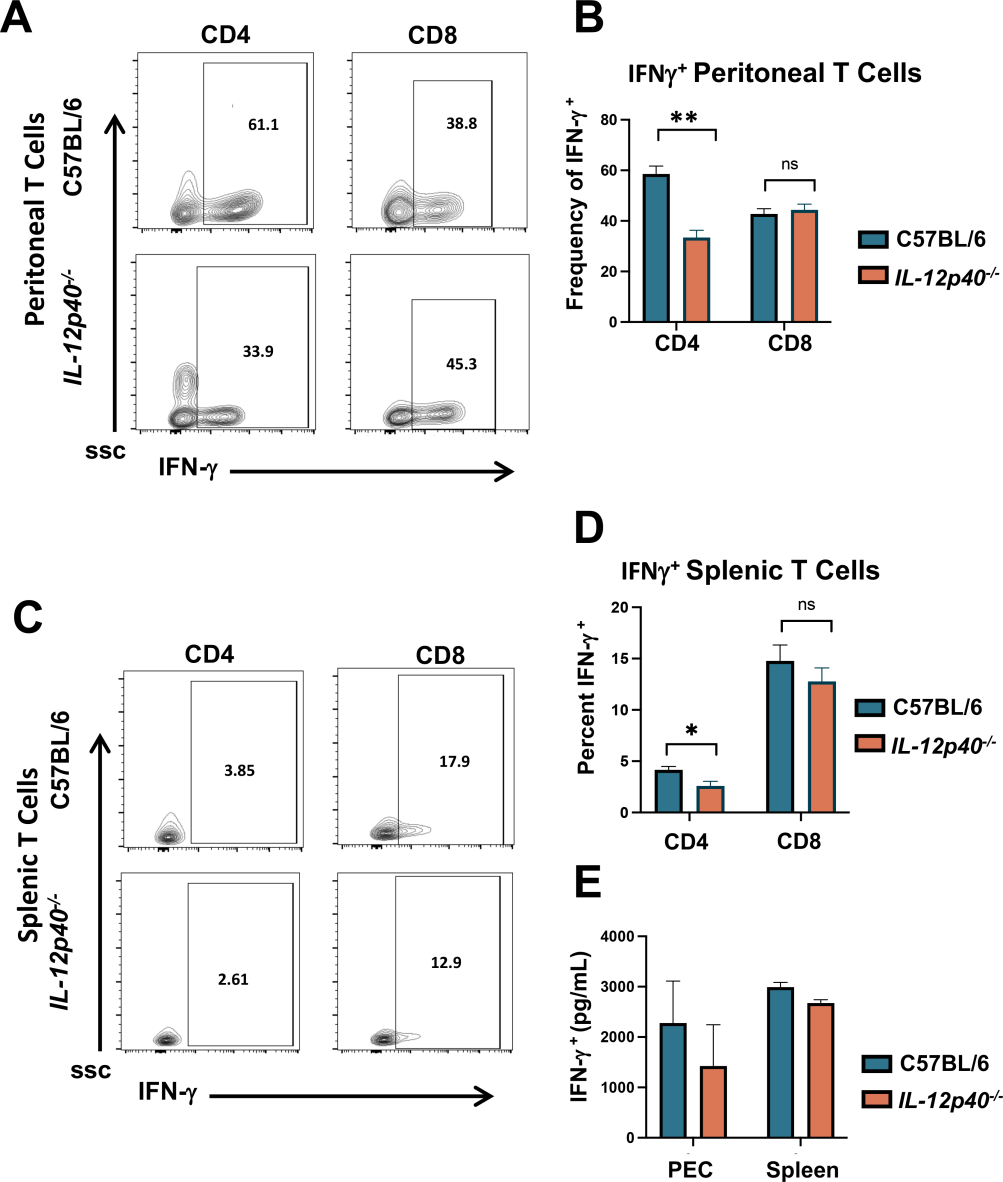
Peritoneal and splenic T cells produce IFN-γ in the absence of IL-12 during OMP infection. C57BL/6 and *IL-12p40^−/−^* mice were inoculated with 10^6^ OMP tachyzoites. At day 7 post-inoculation, PEC and spleens were collected for analysis. (**A**) Representative flow cytometry plots showing IFN-γ expression in T cell subsets from C57BL/6 and *IL-12p40^−/−^* mice after subgating for CD4^+^ and CD8^+^ T cells. Cells were assessed for intracellular IFN-γ expression (x-axis) and side scatter (y-axis) following *in vitro* phorbol 12-myristate 13-acetate (PMA)/ionomycin stimulation. (**B**) Average percent IFN-γ-positive peritoneal CD4^+^ and CD8^+^ T cells. (**C**) Representative flow cytometry plots of splenic T cells and IFN-γ expression from the same mice shown in (**A**) and (**B**). The average percent of splenic T cells expressing IFN-γ is shown in panel D. (**E**) Ex vivo cytokine production by PEC and splenocytes isolated at day 7 post-OMP inoculation. Cells were cultured for 24 hours without further stimulation, and supernatants were subsequently harvested for cytokine ELISA. Un-paired Student’s *t*-test with Welch’s correction and multiple unpaired *t* test were used to analyze the data where *P < 0.05, **P < 0.01, and ns, nonsignificant.

Given the complete IL-12 independence and partial IFN-γ requirement in resistance to OMP, we wanted to determine the state of T cell IFN-γ elicited during infection. We examined intracellular CD4^+^ and CD8^+^ IFN-γ expression in WT and IL-12p40 KO mice 1 week after OMP inoculation. In the peritoneal cavity (Fig. 8A), a large percentage of *IL-12p40^+/+^* CD4^+^ and CD8^+^ cells expressed IFN-γ, with CD4^+^ T cells responding more strongly than the CD8^+^ subset. In IL-12 KO mice (Fig. 8A), IFN-γ expression was retained. However, for CD4^+^ cells, expression was decreased by approximately 50%, while the CD8^+^ IFN-γ response was not affected (Fig. 8B). We also assessed IFN-γin splenic T cells from the same animals (Fig. 8C). In this case, CD4^+^ T cell IFN-γ expression remained close to background levels, although there was a slight but significant decrease in the absence of IL-12p40 (Fig. 8D). The splenic CD8^+^ response differed from that of the CD4^+^ cells, insofar as 13-18% of the cells expressed IFN-γ. Similar to the CD8 cells in the peritoneal cavity, the presence or absence of IL-12p40 did not significantly impact IFN-γ expression (Fig. 8D). We also examined ex vivo cytokine production by PEC and splenocytes from day 7 OMP-infected mice. Cells from both IL-12p40^+/+^ and IL-12p40^−/−^ mice produced large amounts of IFN-γ (Fig. 8E).

Given our observation that we could detect IFN-γ-expressing peritoneal T lymphocytes even in the absence of IL-12, we next asked whether activated inflammatory monocytes might play a role in T cell-dependent protection against OMP. Ly6C/G (GR-1^+^) inflammatory monocytes and neutrophils have long been known to be key effectors in control of the parasite through mechanisms such as IRG-mediated parasitophorous vacuole destruction, iNOS-mediated nitric oxide production, and neutrophil extracellular trap release (35, 72). Therefore, we administered mice anti-Ly6C/G mAb to deplete these cells over the first 14 days of OMP infection (Fig. S2A). Mice depleted of Ly6C/G^+^ cells retained resistance to OMP, unlike *Tcrb^−/−^* mice (Fig. S2B). We conclude that inflammatory monocytes and neutrophils are unlikely to be effectors of protection in this infection model.

### T lymphocyte-dependent IL-10 is required for resistance to OMP

**Fig 9:**
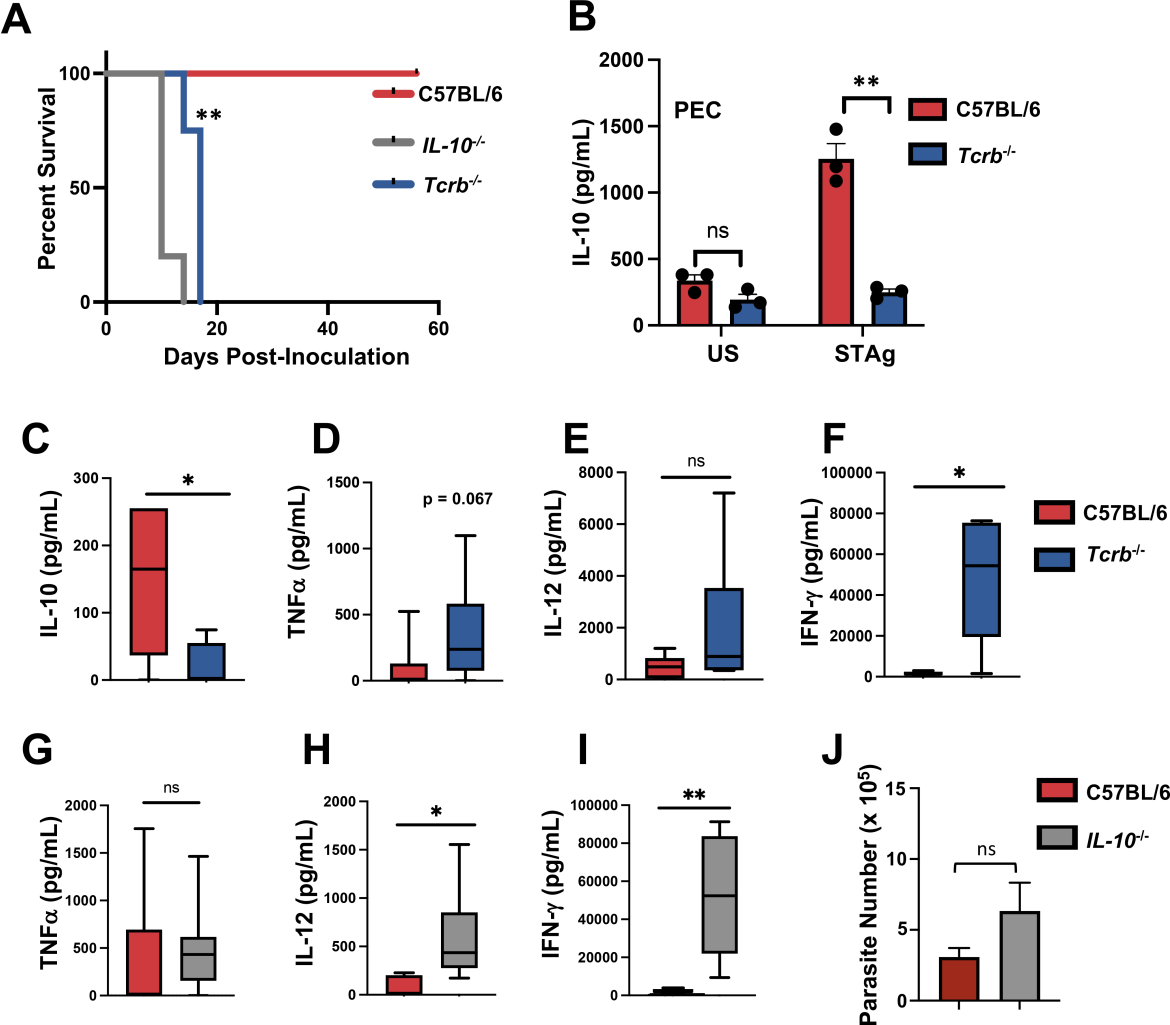
T lymphocyte dependent IL-10 is required for resistance against OMP inoculation. (**A**) OMP parasites were inoculated into *IL-10^−/−^*, *Tcrb^−/−^*, and C57BL/6 animals via i. p. injection and survival monitored. (**B**) D6 OMP PEC from *Tcrb^−/−^* and C57BL/6 animals were left unstimulated (US) or cultured with soluble tachyzoite antigen (STAg), and IL-10 secretion levels were assessed 72 hours later. Sera collected from C57BL/6 and *Tcrb^−/−^* mice at day 15 post-OMP infection was examined for (**C**) IL-10, (**D**) TNFα, (**E**) IL-12, and (**F**) IFN-γ cytokines by ELISA. Sera collected from WT and *IL-10^−/−^* animals at day 12 post-OMP infection was examined for (**G**) TNF-α, (**H**) IL-12, and (**I**) IFN-γ cytokines by ELISA. (**J**) Numbers of OMP tachyzoites in the peritoneal cavities of WT and KO mice 12 days post-infection. Susceptibility study statistics were acquired using Kaplan Meier log-rank tests to analyze the data where **P < 0.01, comparing *IL-10^−/−^* with C57BL/6. Serum ELISAs were the result of two independent studies (*n* = 3 mice per group), and data were analyzed by Mann-Whitney *t*-test, where *P < 0.05 and **P < 0.01.

In an effort to determine the mechanism underlying the T cell requirement for resistance to OMP, we screened a large collection of knockout mice. These strains included *Fasl^gld^* (Fig. S3), perforin^−/−^, gasdermin D^−/−^, and TNFR1/2^−/−^ animals. None proved susceptible to OMP. In marked contrast, animals deficient in IL-10 displayed similar susceptibility survival kinetics as *Tcrb^−/−^* mice following OMP inoculation (Fig. 9A). When cultured D6 PECs were stimulated ex vivo with soluble *T. gondii* antigen (STAg), there was robust IL-10 production in cells from WT animals compared to *Tcrb^−/−^* animals (Fig. 9B). Because of the well-known anti-inflammatory function of IL-10, we examined systemic proinflammatory cytokine levels in WT and *Tcrb^−/−^* animals following OMP inoculation. Levels of circulating IL-10 were significantly lower in the absence of T cells (Fig. 9C). There was an increase in TNFα and IL-12 in *Tcrb^−/−^* mice although neither reached statistical significance (Fig. 9D and E). However, IFN-γ was present at extremely high levels in serum in T cell-deficient animals (Fig. 9F). Histology of the lung, liver, and spleen at peak infection displayed evidence for tissue damage in *Tcrb^−/−^* mice, although there was no significant difference observed between groups (data not shown). To further examine the cytokine profile resulting from OMP infection, we also examined systemic inflammatory cytokines in infected *IL-10^−/−^* animals. Although we did not detect a difference in TNF-α (Fig. 9G), there was a significant increase in the amount of IL-12 and IFN-γ in the absence of IL-10 (Fig. 9H and I) in the serum, although we did not detect a difference in TNFα production (Fig. 9G). This difference in circulating cytokine was evident despite no significant difference in parasite number within the peritoneal cavity (Fig. 9J).

**Fig 10:**
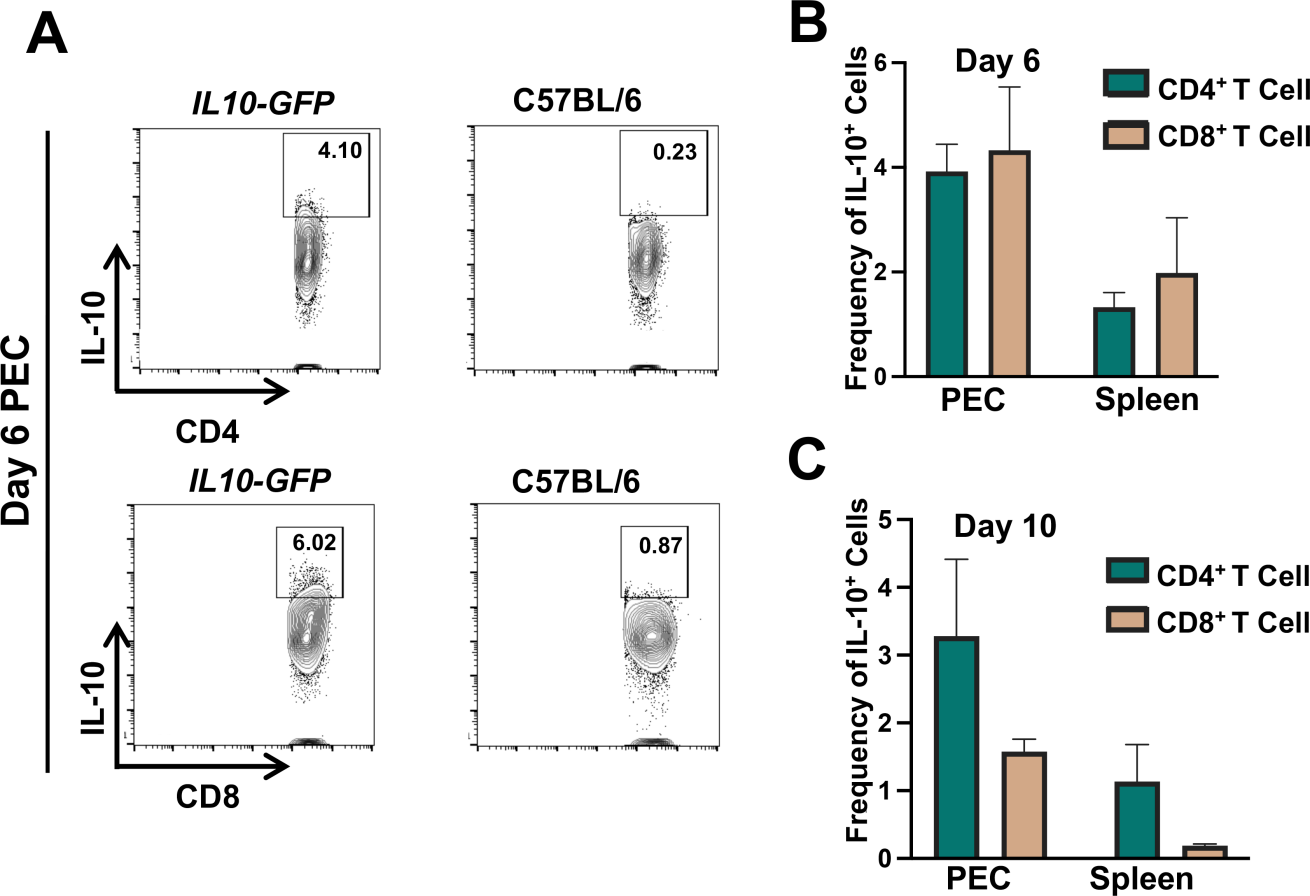
Peritoneal T lymphocytes produce IL-10 in response to OMP inoculation. (**A**) Representative fluoresecence-activated cell sorting (FACS) plots of peritoneal T lymphocyte populations from VertX IL-10 reporter mice alongside C57BL/6 controls. PECs and spleens from VertX IL-10 reporter mice were collected and assessed for GFP expression on αβ T lymphocyte populations at D6 (**B**) and D10 (**C**) after OMP infection. Data shown are average of three independent infections (*n* = 2 mice per group).

To ascertain the cytokine contribution from CD4^+^ and CD8^+^ T lymphocytes during OMP infection, IL-10 reporter animals (VertX; *IL10-GFP*) were employed (Fig. 10A). We found that the VertX mice reported expression of IL-10 in peritoneal cavity CD4^+^ and CD8^+^ T cells, in particular at day 6 post infection (Fig. 10B and C). Notably, expression was significantly less in splenic T cells at the same time point. By day 10, IL-10 expression as reported by green fluorescence protein (GFP) fluorescence was declining in the CD8^+^ population (Fig. 10C).

### GRA24 is an OMP virulence factor in *Tcrb^−/−^* mice

**Fig 11:**
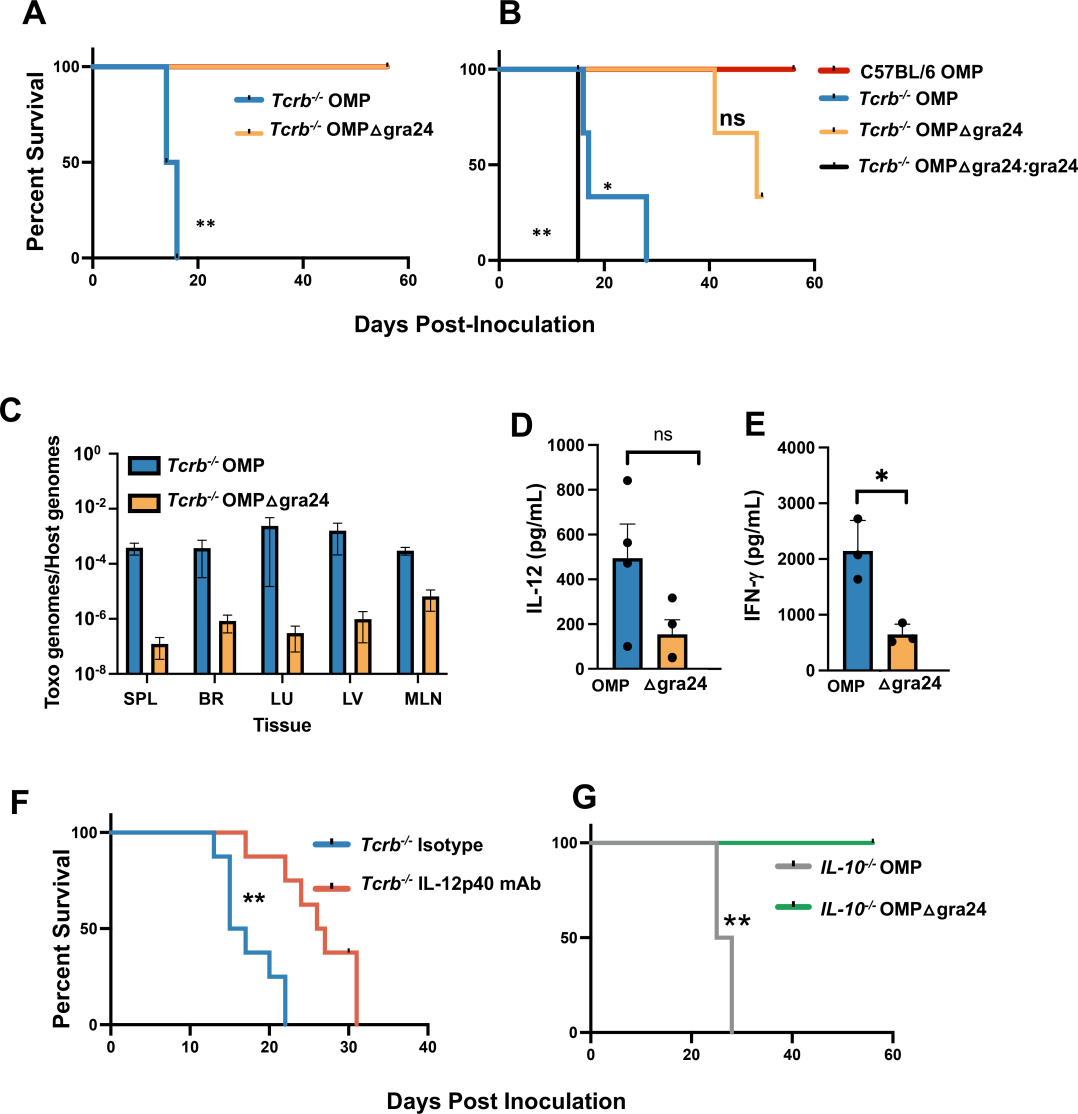
*Toxoplasma* dense granule protein GRA24 acts as a virulence factor in *Tcrb^−/−^* and *IL-10^−/−^* animals. (**A**) Survival of *Tcrb^−/−^* mice following i. p. inoculation of OMP and OMPΔgra24 tachyzoites. (**B**) Survival of *Tcrb^−/−^* mice after i. p. inoculation with OMP, OMPΔgra24, and the complementation mutant OMPΔgra24:gra24. In this experiment, C57BL/6 mice infected with OMP served as controls. Susceptibility studies were repeated three times (*n* = 4 mice per strain) with two representative studies shown. Statistics were acquired using Kaplan Meier log-rank tests to analyze the data where **P < 0.01. ns, no significance comparing *Tcrb^−/−^* OMPΔGRA24 with C57BL/6 OMP. (**C**) Parasite burden assessed by quantitaive PCR (qPCR) of parasite B1 and host arginosuccinate lyase (ASL) genes at day 10 post-infection in *Tcrb^−/−^* mice. Data were quantitated by comparison to B1 and ASL standard amplification curves and are expressed as parasite genomes relative to host genomes. Two independently performed experiments are shown, quantitating levels in spleen (SPL), brain (BR), lung (LU), liver (LV), and mesenteric lymph node (MLN). (**D and E**) PEC collected 10 days post-infection with OMP and OMPΔgra24 in *Tcrb^−/−^* were cultured for 72 hours without further stimulation. Supernatants were assessed by ELISA for (**D**) IL12p40 and (**E**) IFN-γ. (**F**) Survival of *Tcrb^−/−^* mice undergoing mAb-mediated depletion of IL-12p40 relative to IgG isotype control after i. p. inoculation of OMP. (**G**) Survival of *IL-10^−/−^* animals following i. p. inoculation of OMP and OMPΔgra24 tachyzoites. Statistics in (**D**) and (**E**) are the result of unpaired Student’s *t*-tests where **P < 0.01. Susceptibility study statistics in (**F**) and (**G**) were acquired using Kaplan Meier log-rank tests to analyze the data where *P < 0.05.

The *Toxoplasma* dense granule protein GRA24 is a secreted host-directed effector molecule that directly activates p38 mitogen-activated protein kinase (MAPK), resulting in the production of IL-12 and other inflammatory cytokines and chemokines (76, 77). This pathway bypasses signaling mediated through MyD88. We recently reported that GRA24 plays a role in OMP-driven induction of protective immunity in C57BL/6 mice (55). Strikingly, we found here that deletion of GRA24 neutralizes the virulence of OMP in *Tcrb^−/−^* mice (Fig. 11A). In confirmation of this result, the virulence of OMPΔgra24 was restored in a GRA24 complementation mutant (OMPΔgra24:gra24; Fig. 11B). When tissue levels of OMP and OMPΔgra24 DNA were quantitated in day 10-infected mice, we consistently found lower amounts of OMPΔgra24 relative to OMP parasites (Fig. 11C). We also isolated PEC from mice infected with OMP and GRA24-deleted OMP and measured cytokine levels following *in vitro* culture. Although not statistically significant in this experiment, IL-12p40 levels were lower in the absence of GRA24 (Fig. 11D). However, secretion of IFN-γ in PEC cultures was significantly less in the absence of GRA24 (Fig. 11E). The neutralization of virulence through deletion of GRA24 implicated IL-12 as a death driver in OMP-infected Tcrb KO mice. In support of this, IL-12 depletion prolonged time to death in OMP-infected TCRβ-deficient animals (Fig. 11F). Furthermore, IL-10-deficient animals were resistant to OMPΔgra24 even while rapidly succumbing to OMP infection (Fig. 11G). We conclude that the virulence of OMP in a T cell-deficient setting is dependent upon the GRA24 parasite protein that contributes to a lethal proinflammatory cytokine response that is at least partially dependent upon IL-12.

## DISCUSSION

**Fig 12:**
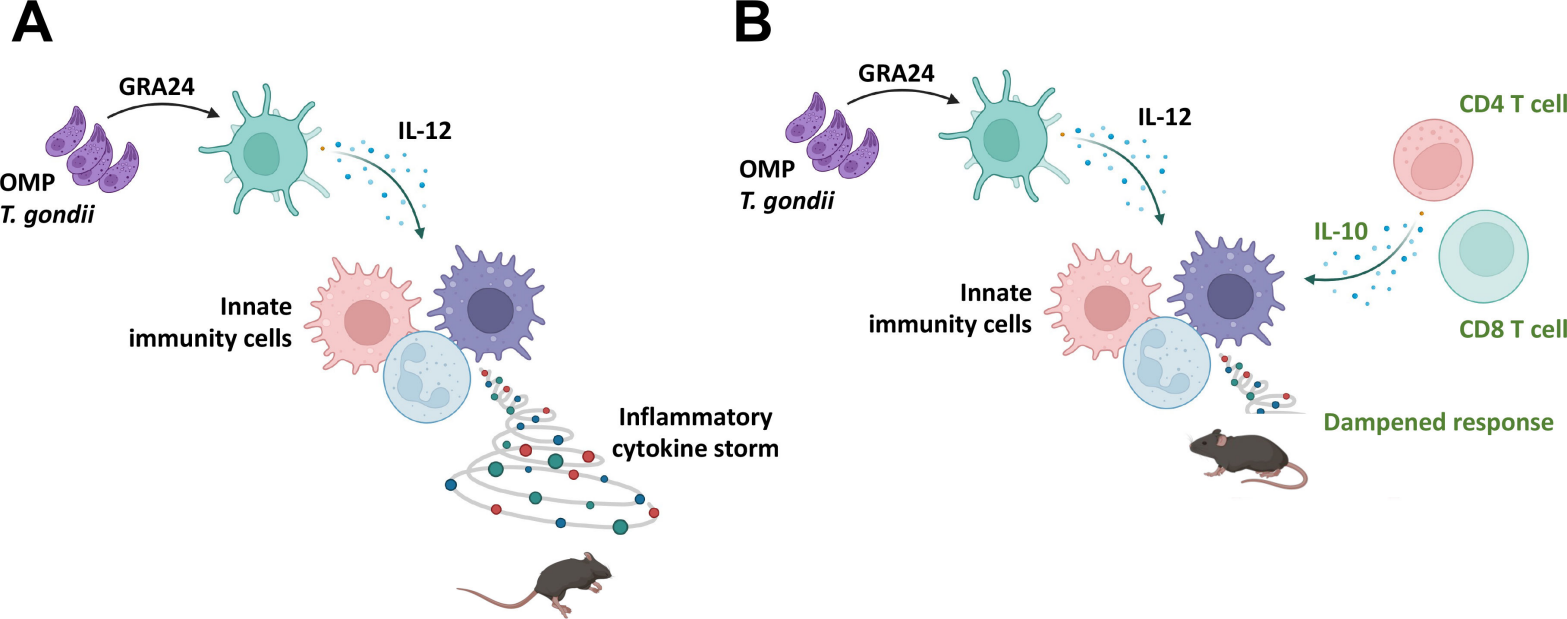
T cell-dependent IL-10 controls the proinflammatory effects of GRA24. (**A**) In the absence of the T cell compartment, GRA24 triggers an innate immune cell-mediated proinflammatory cytokine storm resulting in early death. The function of GRA24 in this context is mediated at least in part through its ability to induce p38 MAPK-dependent IL-12 production. (**B**) In the presence of IL-10-producing CD4^+^ and CD8^+^ T cells, the proinflammatory effects of GRA24 are controlled, and mice survive OMP infection. Created with BioRender.com.

We report here the unexpected finding that after high-dose infection the attenuated Type 1 parasite, OMP, emerges as a virulent *Toxoplasma* strain in the absence of an intact T cell compartment. Both CD4^+^ and CD8^+^ T cells are required for optimal protection, although transgenic knockout and antibody depletion studies together point to CD8^+^ T lymphocytes as the primary protective cell. Interestingly, *IL12p40^−/−^* mice retained full resistance to OMP, indicating that T cell-mediated protection operates independently of this canonical Th1-driving cytokine. We also found that CD8^+^ T cells, and to a lesser extent CD4^+^ T cells, elicited by OMP produced IFN-γ independently of IL-12. Nevertheless, gene knockout studies established only a partial requirement for IFN-γ in resistance to OMP. This is another unexpected insight revealed by OMP insofar as IFN-γ has long been regarded as the major mediator of resistance to *Toxoplasma* during infections with low virulence wild-type strains such as ME49, as well as attenuated temperature-sensitive mutants such as ts-4 (74, 78, 79). We also did not find evidence that Ly6C/G^+^ inflammatory monocytes or neutrophils were involved in resistance to OMP, arguing against these cells as immune effectors. Instead, we uncovered evidence for involvement of T cell-dependent IL-10 in protecting against death, and the lethality of OMP in a T cell-deficient setting was most likely the result of proinflammatory cytokine overproduction. Lastly, the virulence of OMP in TCRβ KO animals depended upon the parasite-dense granule protein GRA24 and its ability to trigger p38 MAPK-dependent proinflammatory cytokines, including IL-12 (summarized in Fig. 12).

We found that peritoneal cavity extracellular tachyzoite levels were higher in *Rag1^−/−^* relative to *Tcrb^−/−^* mice (Fig. 1B vs Fig. 4A). The most straightforward explanation of this result is that immunoglobulins play a role in controlling parasite number. Nevertheless, antibodies seem unlikely to ultimately determine survival because μMT mice lacking B cells were completely resistant to OMP (Fig. 3). This provides further evidence, albeit indirect, that death in *Rag1^−/−^* and in particular *Tcrb^−/−^* mice results from immunopathologic effects, rather than uncontrolled parasite replication.

We note that the results of our studies contrast in some ways with previous related studies. For example, *Rag1^−/−^* mice resist infection with the related uracil-dependent parasite strain cps1-1 (62). In addition, it has been found that IFN-γ KO mice survive cps1-1 infection (50), in contrast to the partial susceptibility reported here. Nevertheless, in addition to using cps1-1, which only replicates at uracil concentrations higher that 32 µM (Fig. 1E), the latter study employed cytokine gene deletion on a BALB/c genetic background rather than the C57BL/6 background used here. Thus, it seems likely that variation between parasite and mouse strains accounts for these differences.

Both antibody depletion and gene knockout experiments lead us to conclude that the combined activity of CD4^+^ and CD8^+^ cells is required for resistance to OMP, with a dominant role for CD8^+^ T cell function. This is consistent with multiple previous studies in other models characterizing protective immunity to *Toxoplasma* (18, 80–84). We report a notable IL-10 contribution from both subsets of αβ T cells; thus, it remains unclear why resistance is predominantly CD8-dependent. One possibility we are examining is that in addition to IL-10, CD8^+^ T cells provide IFN-γ which is required for full resistance to OMP (Fig. 7B).

The cytokine IL-12 has long been considered indispensable for generation of IFN-γ-producing T cells and concomitant protective immunity during *T. gondii* infection (67, 85, 86). Early studies showed that *IL12p40^−/−^* and *Ifng^−/−^* mice succumb equally rapidly following low virulence parasite infection, whereas lymphocyte-deficient severe combined immunodeficiency mice succumbed with delayed kinetics (79, 87). The latter delay was attributed to protective activity of natural killer cells acting as a stopgap mechanism prior to the emergence of adaptive immunity. It is notable that in the present study, we observed that T cell-deficient mice were markedly more susceptible than mice lacking expression of either IFN-γ or most strikingly IL-12.

Batf3-dependent CD8^+^ DC and peripheral CD103^+^ DC have been shown to be an essential IL-12 source during infection (12). Our results uncover an IL-12-independent mechanism of protective immunity. While *Tcrb^−/−^* animals failed to survive OMP infection, *IL-12p40*^*−/−*^ mice were resistant to OMP. We show here that the appearance of IFN-γ-producing T cells does not absolutely require IL-12. For the case of CD4^+^ T cells, we found a partial defect in IFN-γ production in *IL12p40^−/−^* mice, but for CD8^+^ T cells, IFN-γ expression was completely IL-12 independent. Generation of IFN-γ-producing T cells independent of IL-12 is not without precedent. For example, Type 1 IFN can promote early Th1 differentiation during viral infection and after intracellular delivery of bacterial lipopolysaccharide to dendritic cells (88–90). The damage-associated cytokine IL-33 can induce IL-12-independent Th1 effectors that mediate graft vs host disease (91). Other molecules, such as the TNF-related costimulatory molecule CD70, as well as intracellular signaling molecules including Delta 4 Notch-like ligand and T-bet, are capable of inducing Type 1 cytokine responses independently of IL-12 (92–94). Investigations are currently underway to determine which, if any, of these factors are involved in the T cell-dependent protective immune response to OMP *T. gondii*.

Our studies reveal that *Toxoplasma*-dense granule protein GRA24 acts as an OMP virulence factor in a T cell-deficient setting. This parasite effector protein is transported across the parasitophorous vacuole membrane in a manner involving several parasite molecules including Myr proteins, ROP17, and TgASP5 (95). In the host cell, GRA24 translocates to the nucleus and directly activates p38 MAPK in a mechanism involving autophosphorylation (76). Phosphorylation of p38 MAPK results in induction of IL-12 in macrophages and dendritic cells and contributes to induction of protective immunity in wild-type mice (55, 77). Here, we found that IL-12 depletion results in partial rather than full rescue of OMP-infected *Tcrb^−/−^* mice. However, in addition to IL-12, GRA24 upregulates several other proinflammatory cytokines and chemokines, including IL-6, TNF, *MCP-1*, *CCL5*, *CXCL1*, and CXCL10 that could contribute to the lethal outcome of infection (76). Thus, a likely scenario is that OMP triggers a GRA24-dependent cytokine storm that results in host mortality in *Tcrb^−/−^* mice. This would be consistent with our observation that despite uniform lethality in *Tcrb^−/−^* mice, OMP levels in tissues are overall very low (Fig. 4 and 11C). We note, nevertheless, that tissue levels of *OMPΔ*GRA24 parasites were lower than OMP *Toxoplasma* (Fig. 11C)*.* Superficially, this seems paradoxical since *GRA24-*driven *IL-12* would be expected to drive a strong *Th1* response to more effectively control *T. gondii*. However, it is possible that a fulminating dysregulated proinflammatory cytokine response interferes with the ability to control the parasite in a precisely regulated manner. Another possibility is that an absent GRA24-mediated IL-12/Th1 response results in a reciprocal increase in the antibody response which our data suggest may control tachyzoite number. These areas are ripe for future investigation.

We report here that both *IL-10^−/−^* and *Stat1^−/−-^* mice are equally susceptible to OMP, an unexpected result given the opposing inflammatory effects of these immune mediators. However, this result recalls older studies in which IL-10 KO and IFN-*γ* KO mice succumbed to low virulence ME49 infection with similar kinetics (79, 96). In those cases, death was attributed to proinflammatory cytokine overproduction in the absence of IL-10 and parasite-mediated tissue destruction in the absence of IFN-*γ*. It is also notable that a recent study provides evidence that STAT1 is required for T cell production of IL-10 during *Toxoplasma* infection, which would be consistent with our results (47). The extent to which STAT1 controls OMP replication *in vivo* is currently under investigation.

The present work reveals several heretofore unrecognized facets of the immune response induced by *Toxoplasma*. Nevertheless, a question arises regarding the relevance of our findings to immune responses to naturally occurring *Toxoplasma* strains, insofar as we employed here a laboratory-engineered low virulence parasite. However, the IL-12-independent immunity we found here may have relevance to human infectious diseases where IL-12 appears to be dispensable, at least to a certain extent (97). It will be of interest to examine immune responses triggered by other naturally occurring low virulence *T. gondii* strains, in addition to Type 2 parasites that have been extensively studied, to determine the extent to which such strains elicit OMP-like responses. Regardless, from an immunological perspective, our studies reveal new and unexpected insights into how the immune system responds to infectious challenge with *Toxoplasma* and possibly other microbial pathogens.

## MATERIALS AND METHODS

### Mice

Mice were purchased from The Jackson Laboratory (Bar Harbor, ME, USA). The strains used in this study were C57BL/6J (*B6*, Stock #000664), B6.129S1-*Il12b^tm1Jm^*/J (*IL12p40^−/−^*, Stock # 002693), B6.129P2-*B2m^tm1Unc^*/DcrJ (*β2m*^*-/-*^, Stock #002087), B6.129S2-*H2^dlAb1-Ea^*/J (*Aβ*^*−/−*^, Stock #003584), B6.129P2-*Tcrb^tm1Mom^*/J (*TCRb^−/−^*, *Stock #002118)*, B6.129S7-*Rag1^tm1Mom^*/J (*Rag1^−/−^*, Stock #002216), B6.129S7-*Ifng^tm1Ts^*/J(*IFNg^−/−^*, Stock #00287), *Tcrd^tm1Mom^*/J (*Tcrd^−/−^*, Stock #002122), B10.129S2(B6)-*Ighm^tm1Cgn^*/J (*MuMT^−/−^*, Stock #002249), B6Smn.C3-Fas^gld^/J (*Fasl gld*, stock #001021), B6.129P2(SJL)-*Myd88^tm1.1Defr^*/J (*Myd88^−/−^*, Stock #009088), B6(Cg)-Il10^tm1.1Karp/^J (VertX, Stock #014530), and B6.129S4-Ifng^tm3.1Lky^/J (GREAT, Stock #017581). Mice were maintained in the Department of Biology Animal Research Facility at the University of New Mexico. Animals involved in these studies ranged from 6 to 9 weeks of age. Both female and male mice were used throughout.

### Parasites and infections

The design and construction of Type I uracil auxotroph *T. gondii* parasite strain *∆ompdc∆up* (herein designated OMP) have been previously described (61). This *Toxoplasma* strain lacks genes encoding orotidine-5′-monophosphate decarboxylase and uridine phosphorylase, required for *de novo* pyrimidine synthesis and salvage, respectively. OMP tachyzoites were maintained *in vitro* by serial passage in human foreskin fibroblast monolayers in media supplemented with 300 µM exogenous uracil. Generation of the GRA24-deleted OMP strain and the GRA24 complementation mutant was previously described (55). Parallel cultures of parasites were passaged one a month using media lacking supplemental uracil to ensure persistence of the uracil auxotroph phenotype. Parasite cultures were tested for *Mycoplasma* contamination every 3 months (MycoProbe Detection Assay; R & D Systems, Minneapolis, MN). Mice were infected with 1–2 × 10^6^ tachyzoites via intraperitoneal injection.

### Immunofluorescence assay

Cells (10^5^) from peritoneal washouts were cytospun (2 min, 700 rpm) onto glass coverslips, then fixed for 20 min in 3.7% paraformaldehyde in a 24-well tissue culture plate. After washing in phosphate-buffered saline (PBS), cells were blocked for 30 min at room temperature (RT) with 5% normal mouse serum in PBS containing 0.1% saponin (permeabilization buffer; PB). The cells were subsequently incubated (60 min, RT) with goat anti-*Toxoplasma* FITC-conjugated antibody (Invitrogen, Carlsbad, CA; catalogue number #PA17254) in PB with 5% bovine serum albumin (Sigma-Aldrich, Burlington, MA), washed, and mounted onto glass microscope slides with ProLong Diamond Antifade Mountant containing DAPI (ThermoFisher Scientific, Waltham, MA; catalogue # P36962). Slides were allowed to cure for 24 hours prior to image collection. Imaging was carried out using a BX53 fluorescence microscope and DP manager software (Olympus, Waltham, MA).

### Uracil rescue profile of cps1-1 and OMP

The intracellular parasite growth rate was measured by scoring tachyzoites per vacuole 32 hours post-infection. Freshly lysed OMP and cps1-1 tachyzoites cultured in high uracil were filtered through Nuclepore membranes, and tachyzoites were washed twice in PBS to remove uracil. Replicate human foreskin fibroblasts (HFF) monolayers were infected with OMP or cps1-1 for 1 hour at a multiplicity of infection of 0.1 in the absence of uracil, monolayers were rinsed in PBS to remove uninvaded tachyzoites, and duplicate infected HFF’s were incubated in infection medium containing various amounts of uracil. At 32 hours post-infection, the number of tachyzoites per vacuole was scored from 50 randomly selected vacuoles. The average number of tachyzoites per vacuole at each uracil concentration was determined in three independent experiments.

### Cell culture and ELISA

Single-cell suspensions were cultured ex vivo at 2 × 10^6^ cells/mL in complete DMEM (cDMEM) consisting of 10% bovine growth serum (HyClone, Logan, UT, USA), 1% non-essential amino acids (Thermo Fisher Scientific, Waltham, MA, USA), 30 mM HEPES (Thermo Fisher Scientific), 1 mM sodium pyruvate (Thermo Fisher Scientific), 100 U/mL penicillin + 0.1 mg/mL streptomycin (Thermo Fisher Scientific), and 50 mM 2-mercaptoethanol (Sigma Aldrich, St. Louis, MO, USA). Cells were cultured in media alone or stimulated with soluble tachyzoite antigen (STAg, 50 mg/mL). The resulting cell culture supernatants were collected 72–96 hours post-plating. IL-12p40 and IFN-γ levels were quantified by ELISA following the manufacturer’s recommendations (Invitrogen, Waltham, MA, USA). STAg was prepared by sonicating RH strain tachyzoites at 0°C in the presence of protease inhibitors, followed by centrifugation at 10,000 × *g* as previously described (55). Supernatant was collected, dialyzed into PBS, and stored in aliquots at −80°C. Endotoxin levels in STAg were determined by commercial assay (Pierce, ThermoFisher #A39552) and found to be present at <0.25 EU/mL.

### Serum analysis

Sera were collected via cardiac puncture with a 21 Ga needle. Samples were rested for 0.5 hours at room temperature to allow for clot formation. Serum was then centrifuged 10,000 × *g* for 10 min at 4°C, and supernatant was collected for ELISA following the manufacturer’s recommendations (Invitrogen, Waltham, MA, USA).

### Flow cytometry

To study IFN-γ production, cells (1 × 10^6^) were stimulated in 1 mL cDMEM containing 50 ng/mL phorbol 12-myristate 13-acetate (PMA; Sigma Aldrich), 5 mg/mL ionomycin (Alfa Aesar, Tewksbury, MA, USA), and 10 mg/mL Brefeldin A (Biolegend, San Diego, CA, USA, Catalog #420601) for 4 hours at 37°C. Single-cell suspensions were subsequently incubated with Zombie Aqua dye (Zombie Aqua Fixable Viability Kit; Biolegend, Catalog #423101) for 5 min in PBS to stain dead cells. PMA/ionomycin stimulation was not employed in studies involving cells from cytokine reporter animals.

Profiling of cells in the peritoneal cavity was accomplished following collection via a 10 mL PBS flush of the cavity using a 21 Ga needle. PEC were rinsed with FACS buffer (1% BSA, 0.1% sodium azide, PBS), and cell surface markers were labeled with primary antibodies including anti-F4/80 Fluor BV711 (Biolegend, Catalog #123147), anti-Ly6G Fluor PerCP-Cy5.5 (Biolegend, Catalog #127615), anti-CD11b Fluor PE (Biolegend, Catalog #101207), anti-CD11c Fluor PE Dazzle (Biolegend, Catalog #117347), anti-MHCII Fluor AF647 (Biolegend, Catalog #107617), anti-CD5 Fluor PerCP (Biolegend, Catalog #100616), and anti-B220 BV421 (Biolegend, Catalog #103239). T cell surface markers were stained using anti-TCRβ Fluor ACP-Cy7 (Biolegend, Catalog #109220), anti-CD4 Fluor PerCP-Cy5.5 (Biolegend, Catalog #100434), anti-CD8 Fluor PE-Cy7 (Biolegend, Catalog #100722), anti-CD62L Fluor PE (Biolegend, Catalog #104408), anti-CD44 Fluor Pacific Blue (Biolegend, Catalog #103020), and anti-CD69 Fluor FITC (Biolegend, Catalog #644813).

After surface marker staining, cells were rinsed, fixed in 3.7% paraformaldehyde in PBS (15 min at room temperature), washed, and re-suspended in FACS buffer for analysis. For intracellular and nuclear staining, cells were rinsed and incubated overnight in 1 mL of Fixation/Permeabilization buffer (FoxP3/Transcription Factor Staining Kit; eBioscience, Waltham, MA, USA). The following day, samples were rinsed with 2 mL of Fixation/Permeabilization buffer prior to staining intracellular and nuclear proteins in permeabilization buffer for 1 hour at 4°C. Intracellular and nuclear markers were labeled using primary antibodies, including anti-T-bet APC (Biolegend, Catalog #644814), anti-Ki67 FITC (Biolegend, Catalog #151212), or anti-IFN-γ (Biolegend, Catalog #505808). After 1 hour, samples were washed and re-suspended in FACS buffer prior to analysis. Samples were run on an Attune NXT, five laser flow cytometer (ThermoFisher Scientific), and the data were analyzed using FlowJo v.10 software (FlowJo).

### *In vivo* mAb-mediated depletion

Mice were intraperitoneally injected with 0.2 mg anti-CD4 monoclonal antibody (BioXCell, Lebanon, NH, USA, Catalog #BE0003-1, Clone: GK1.5), anti-CD8^+^ monoclonal antibody (BioXCell, Lebanon, NH, USA, Catalog #BE0061, Clone: 2.43), 0.2 mg anti-GR-1 monoclonal antibody (BioXCell, Lebanon, NH, USA, Catalog #BE0075, Clone: RB6-8C5), 0.4 mg anti-IL12p40 monoclonal antibody (BioXCell, Lebanon, NH, USA, Catalog #BE0051), or 0.2 or 0.4 mg rat gamma globulin (Jackson Immunoresearch, West Grove, PA, USA, Catalog #012–000-002) beginning 48 hours prior to *T. gondii* infection and continuing every 48 hours for the duration of the experiment. Confirmation of the effective depletion of respective cell types was ascertained by FACS staining of PEC collected from mice after receiving two intraperitoneal injections of monoclonal antibodies.

### PCR quantitation of parasite and host DNA

Relative levels of host and parasite DNA in tissues from infected (10^6^, i. p., day 12 post-inoculation) mice were determined as described in detail elsewhere (68). Briefly, tissue DNA was isolated and subject to real-time qPCR of the host arginosuccinate lyase gene and the multi-copy parasite B1 gene. Resulting Cq values were converted to respective nanogram amounts of host and parasite DNA by quantitative comparison to Cq values generated by amplification of standard amounts of purified mouse and tachyzoite DNA. Relative genome number was calculated assuming a mouse genome size approximately 34x greater than the *T. gondii* genome size. The applied threshold of discrimination for Cq values was 37 cycles. Amplification reactions were accomplished by employing a BioRad CFX96 RealTime System C1000 Touch thermal cycler.

### Statistical analyses

Statistical analyses were performed using GraphPad Prism v.8 (GraphPad, La Jolla, CA). Two-tailed Student’s *t*-tests were used to compare normally distributed data with only two groups. A Student’s *t*-test with Welch’s correction was also used to compare data sets with unequal variances, and a Mann Whitney test was used to compare data sets that were not normally distributed. A one-way ANOVA with Tukey multiple comparisons post-test was used to compare three or more normally distributed groups. The significance of mouse survival curves was assessed using a Kaplan Meier curve and Log-rank test. A confidence interval of 95% (a = 0.05) was used as the cut-off to denote significant changes between groups.

## SUPPLEMENTAL MATERIAL

Fig. S1 mbio.01455-24-s0001.tif Expression of CD44 and CD62L in splenic T cells of OMP-infected mice.

Fig. S2 mbio.01455-24-s0002.tif Protective immunity against OMP does not require Ly6C/G+ inflammatory monocytes.

Fig. S3 mbio.01455-24-s0003.tif Protective immunity against OMP does not require Fas ligand.

ASM does not own the copyrights to Supplemental Material that may be linked to, or accessed through, an article. The authors have granted ASM a non-exclusive, world-wide license to publish the Supplemental Material files. Please contact the corresponding author directly for reuse.
