# Supplementary figures and images for: T lymphocyte-dependent IL-10 down-regulates a cytokine storm driven by Toxoplasma gondii GRA24
Source: mBio. 2024 Oct 23;15(11):e01455-24. doi: 10.1128/mbio.01455-24 (PMC11559025; doi:10.1128/mbio.01455-24)

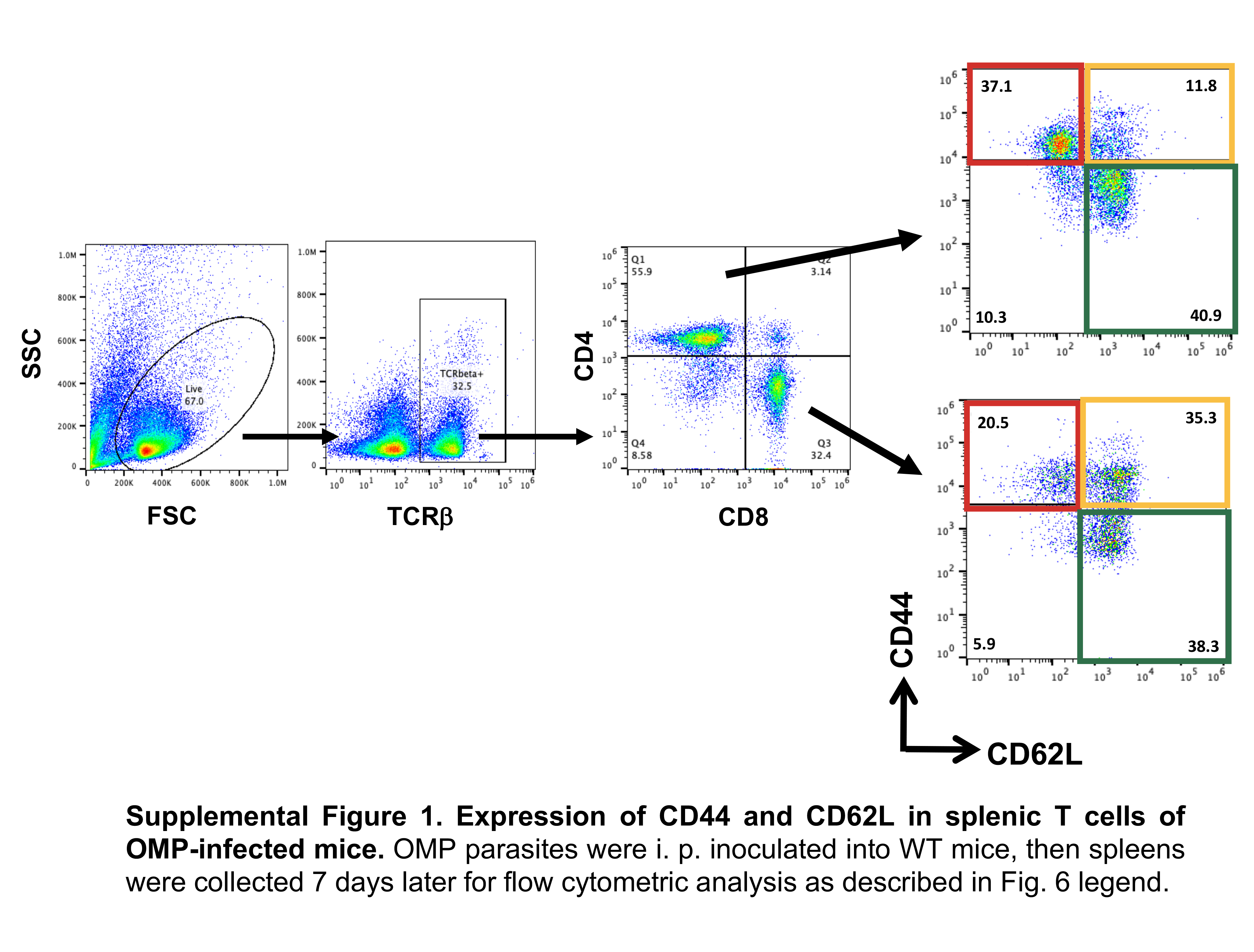

Supplement: Fig. S1 — Expression of CD44 and CD62L in splenic T cells of OMP-infected mice. [file mbio.01455-24-s0001.tif]

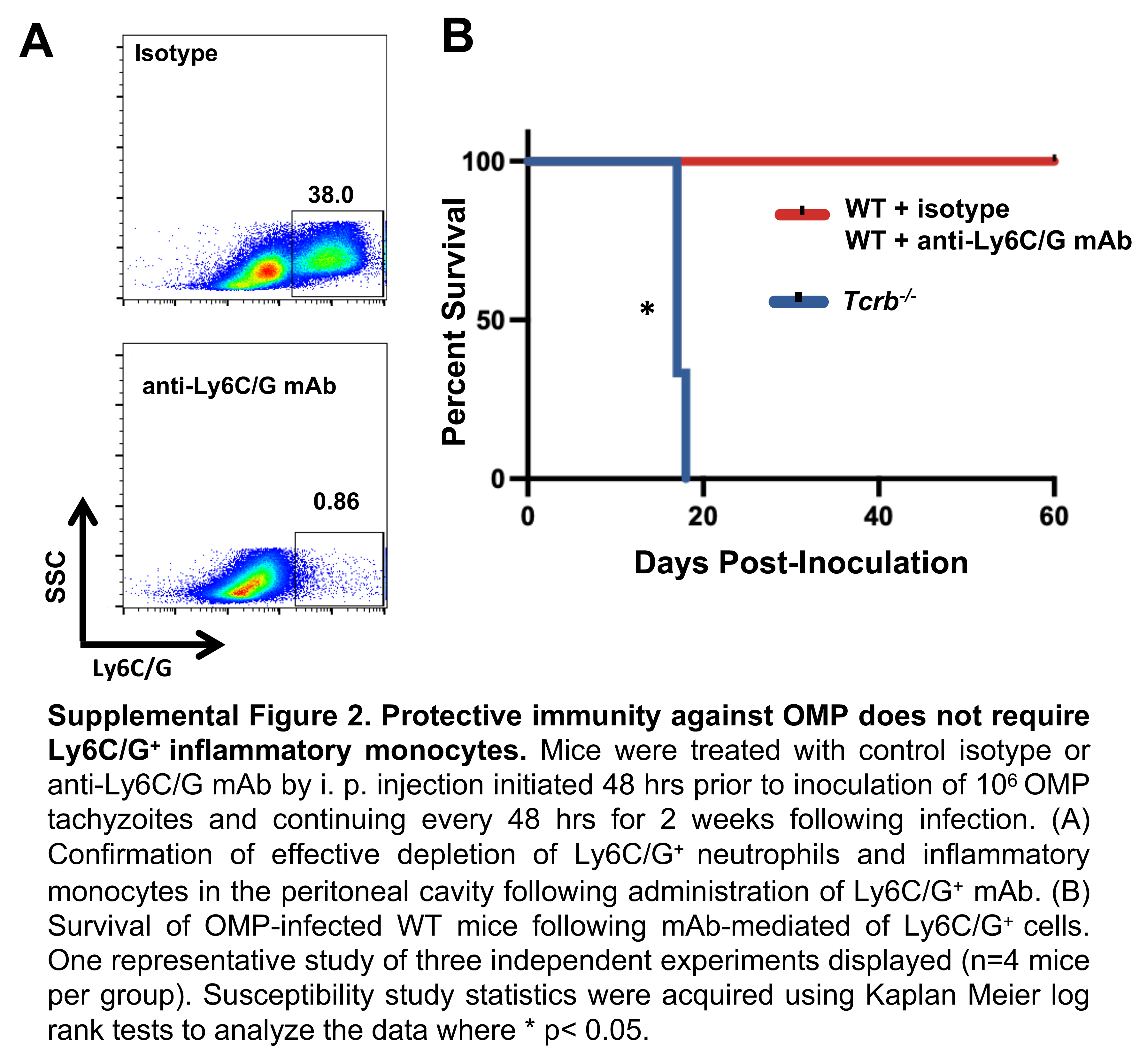

Supplement: Fig. S2 — Protective immunity against OMP does not require Ly6C/G+ inflammatory monocytes. [file mbio.01455-24-s0002.tif]

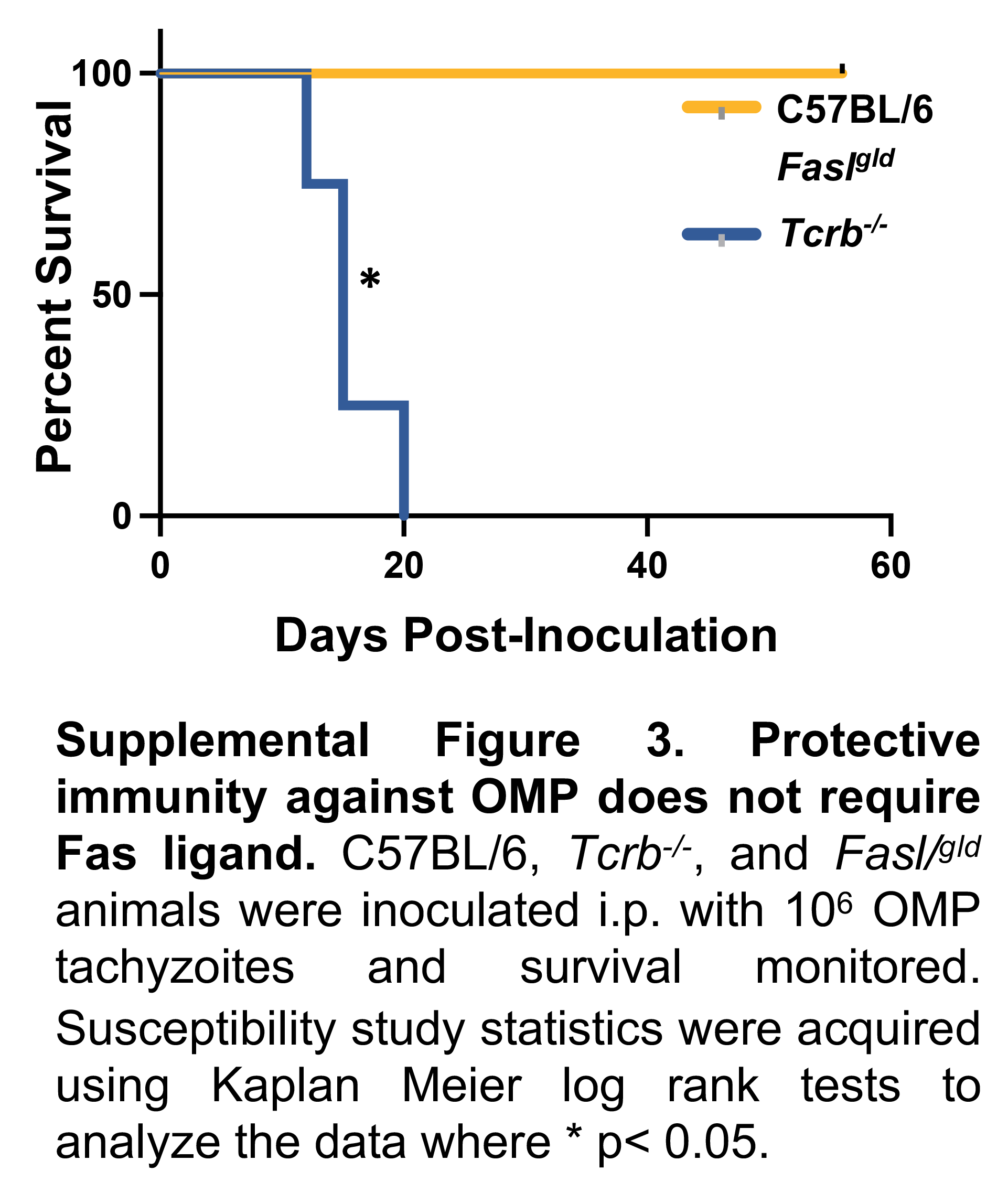

Supplement: Fig. S3 — Protective immunity against OMP does not require Fas ligand. [file mbio.01455-24-s0003.tif]
